# Association between food insecurity and key metabolic risk factors for diet-sensitive non-communicable diseases in sub-Saharan Africa: a systematic review and meta-analysis

**DOI:** 10.1038/s41598-021-84344-0

**Published:** 2021-03-04

**Authors:** Sphamandla Josias Nkambule, Indres Moodley, Desmond Kuupiel, Tivani P. Mashamba-Thompson

**Affiliations:** 1 Department of Public Health Medicine, School of Nursing and Public Health, University of KwaZulu-Natal, Durban, South Africa; 2 Faculty of Health Sciences, University of Pretoria, Pretoria, Gauteng Province South Africa

**Keywords:** Biomarkers, Risk factors

## Abstract

In previous studies, food insecurity has been hypothesised to promote the prevalence of metabolic risk factors on the causal pathway to diet-sensitive non-communicable diseases (NCDs). This systematic review and meta-analysis aimed to determine the associations between food insecurity and key metabolic risk factors on the causal pathway to diet-sensitive NCDs and estimate the prevalence of key metabolic risk factors among the food-insecure patients in sub-Saharan Africa. This study was guided by the *Centre for Reviews and Dissemination* (CRD) guidelines for undertaking systematic reviews in healthcare. The following databases were searched for relevant literature: PubMed, EBSCOhost (CINAHL with full text, Health Source - Nursing, MedLine). Epidemiological studies published between January 2015 and June 2019, assessing the associations between food insecurity and metabolic risk outcomes in sub-Saharan African populations, were selected for inclusion. Meta-analysis was performed with DerSimonian-Laird’s random-effect model at 95% confidence intervals (CIs). The I^2^ statistics reported the degree of heterogeneity between studies. Publication bias was assessed by visual inspection of the funnel plots for asymmetry, and sensitivity analyses were performed to assess the meta-analysis results’ stability. The Mixed Methods Appraisal Tool (MMAT) – Version 2018 was used to appraise included studies critically. The initial searches yielded 11,803 articles, 22 cross-sectional studies were eligible for inclusion, presenting data from 26,609 (46.8% males) food-insecure participants, with 11,545 (42.1% males) reported prevalence of metabolic risk factors. Of the 22 included studies, we identified strong evidence of an adverse association between food insecurity and key metabolic risk factors for diet-sensitive NCDs, based on 20 studies. The meta-analysis showed a significantly high pooled prevalence estimate of key metabolic risk factors among food-insecure participants at 41.8% (95% CI: 33.2% to 50.8%, I^2^ = 99.5% *p*-value < 0.00) derived from 14 studies. The most prevalent type of metabolic risk factors was dyslipidaemia 27.6% (95% CI: 6.5% to 54.9%), hypertension 24.7% (95% CI: 15.6% to 35.1%), and overweight 15.8% (95% CI: 10.6% to 21.7%). Notably, the prevalence estimates of these metabolic risk factors were considerably more frequent in females than males. In this systematic review and meta-analysis, exposure to food insecurity was adversely associated with a wide spectrum of key metabolic risk factors, such as obesity, dyslipidaemia, hypertension, underweight, and overweight. These findings highlight the need to address food insecurity as an integral part of diet-sensitive NCDs prevention programmes. Further, these findings should guide recommendations on the initiation of food insecurity status screening and treatment in clinical settings as a basic, cost-effective tool in the practice of preventive medicine in sub-Saharan Africa.

PROSPERO registration number: PROSPERO 2019 CRD42019136638.

## Introduction

Metabolic risk factors for diet-sensitive non-communicable diseases (NCDs) are accelerating rapidly and advancing across countries. Globally, this results in a substantial morbidity and mortality burden linked to diet-sensitive NCDs, such as diabetes and cardiovascular diseases^1,2^. In 2017, metabolic risk factors claimed an estimated 10.4 million deaths and approximately 218 million disability-adjusted life-years (DALYs)^3^. Thus, due to their substantial contribution to the global disease burden—as well as rapidly increasing trends, and variable patterns across countries—has prompted researchers to investigate factors driving the prevalence of metabolic risk factors. One such factor is food insecurity, defined as the lack of access to nutritionally adequate foods caused by poverty and other socio-economic stressors^4–7^.

According to the World Health Organization (WHO) and the United Nations General Assembly, among other professional organisations, food insecurity has emerged as a global health crisis and an important proximate driver of the prevalence of metabolic risk factors on the causal pathway to diet-sensitive NCDs^8–10^. This premise stems from the realisation that exposure to food insecurity results in compensatory behaviours, which often include less intake of fruit and vegetables^11^, skipping meals, or reducing portion sizes^12^, and overconsumption of low-cost foods that are high in calories yet deficient in nutritional value^13^. Over time, these compensatory behaviours, perceived as buffers against food insecurity, may lead to malnutrition, which, in turn, initiates a cascade of adverse metabolic profile that ultimately precipitate diet-sensitive NCDs^8,14,15^.

However, accumulating evidence on the association between food insecurity and metabolic risk outcomes have yielded conflicting evidence of associations, especially for children and adult males^16^. In contrast, more persuasive evidence of an antagonistic relationship was found in women from high-income countries^13,17^. Evidence of these associations in resources-limited settings with high levels of food insecurity such as the sub-Saharan African countries has not been systematically reviewed or meta-analysed. For these reasons, it does not provide sufficient integrated, evidence-based, and comprehensive information about the distribution of specific key metabolic risk factors among the populace confronted with food insecurity in the region.

Understanding trends and patterns of specific key metabolic risk factors among those confronted with food insecurity is vital for both public health and clinical management of diet-sensitive NCDs. Therefore, we conducted a systematic review and meta-analysis to determine the associations between food insecurity and key metabolic risk factors for diet-sensitive NCDs and to estimate the prevalence of these metabolic risk factors among the food-insecure patients in sub-Saharan Africa. It is anticipated that the study findings may serve as baseline data for setting priorities, designing interventions to reduce associated morbidity and mortality, and strengthening the basis for policy action and recommendations for future research.

## Methodology

### Study design

The study protocol was developed and registered before the study’s start with the International Prospective Register of Systematic Reviews (PROSPERO; CRD42019136638), and the published methodology made available for public comments via the link below: https://www.crd.york.ac.uk/prospero/display_record.php?ID=CRD42019136638

The present systematic review and meta-analysis were conducted following the principles recommended in the *Centre for Reviews and Dissemination* (CRD) guidance for undertaking systematic reviews in healthcare^18^, and the *Meta-analysis Of Observational Studies in Epidemiology* (MOOSE) guidelines for design and implementation^19^. The reporting was aligned to the Preferred Reporting Items for Systematic Reviews and Meta-Analyses (PRISMA) guidelines^20^ to ensure all necessary seven steps have been followed. The completed PRISMA checklist is provided as supplementary material (Supplementary Appendix No. 2).

In this review, we first sought to identify the specific spectrum of key metabolic risk factors associated with food insecurity, by systematically reviewing current peer-reviewed epidemiological studies conducted in sub-Saharan Africa, assessing the associations between food insecurity and key metabolic risk factors on the causal pathway to diet-sensitive non-communicable diseases (NCDs). Secondly, after identifying the spectrum of key metabolic risk factors associated with food insecurity, we sought to assess the prevalence of these metabolic risk factors among the food-insecure patients in sub-Saharan Africa.

Lastly, to assess study methodological quality (or risk of bias) of all included studies, we used the adopted Mixed Methods Appraisal Tool (MMAT)—Version 2018^21^, as it permits for appraising the quality of quantitative non-randomized studies^22,23^.

#### Step 1: Identifying the research question

To determine our research question's eligibility for a systematic review project, we applied the PEO (Population, Exposure, and Outcomes) nomenclature for systematic reviews^24^, as illustrated in Table 1.

**Table 1:** PEO framework for determining the eligibility of the research question.

Criteria	Determinant
Population	Human participants of all-age groups residing in sub-Saharan Africa, both genders, and regardless of their ethnic background
Exposure	Food Insecurity (FI)* (Independent Variable) experienced at either household or individual level
Outcomes	Diagnosed with any of the following key metabolic risk factors for diet-sensitive NCDs per the international diagnostic criteria for metabolic syndromes (MetS) such as NCEP-ATPIII (2001), International Diabetes Foundation (IDF 2005), AHA/NHLBI criteria (2004), and any other measures in line with the World Health Organization criteria (1998). Outcomes of interest are: 1. Obesity 2. Dyslipidaemia 3. Hypertension 4. Underweight 5. Overweight 6. Others (as reported per study)

*Detailed list of essential tools used to define/ or ascertain food insecurity as an independent variable, accompanying theoretical minimum risk exposure level see Supplementary Appendix No. 1 Table 1.

Definition of review outcomes reported assessment beyond general health self-report and the diagnostic criteria for identifying metabolic risk factors for this study see Supplementary Appendix No. 1 Table 2.

The main research question: What are the associations between food insecurity and key metabolic risk factors for diet-sensitive NCDs in the sub-Saharan African population?

#### Step 2: Eligibility criteria

The eligibility criteria were developed according to the relevant elements of the PEO-T (Time) framework guidance for undertaking a systematic review, to ensure that the research question's boundaries are clearly defined. Eligible articles were included after two reviewers had independently, reproducibly, and systematically evaluated them and met the inclusion criteria, as illustrated in Table 2.

**Table 2:** Eligibility criteria according to the PEO-T nomenclature criteria.

Criteria	Inclusions	Exclusions
Study design	Only original and quantitative study design that are peer-reviewed cross-sectional studies conducted in sub-Saharan Africa by trained personnel, in any publication language	Qualitative study design, and systematic reviews, literature reviews, mini-reviews and cross-sectional studies conducted outside of Africa
Population	Studies conducted among sub-Saharan African populations Participants of any age group and sex that did not mix the age groups for analysis Any ethnicity, culture, or race	Research studies conducted among people outside of sub-Saharan Africa Participants with coexisting medical conditions
Exposure	Studies reporting a measure of food insecurity or food insufficiency as an independent variable A measure of food insecurity beyond receiving government-sponsored nutritional benefits/ or living in an area designated as a food desert Food insecurity evaluated at the household level or individual level	Research studies reporting on voluntary food restriction, as in cases of anorexia/Nervosa
Outcomes	Studies reporting at least one of the key metabolic risk factors as a dependent variable Diagnosed with key metabolic risk factors per any of the international diagnostic criteria for MetS: NCEP-ATP III, IDF, or AHA/NHLBI criteria or any other measures in line with the World Health Organization criteria (1998)	Research studies reporting on the outcome as the result of infectious agents None validated outcome measures
Time	Complete peer-reviewed original research studies, published between January 2015 and June 2019	Studies published before January 2015

#### Step 3: Identifying relevant studies

##### (a) Literature search and search strategy

Identification of eligible STUDIES for inclusion in this review was through a comprehensive and reproducible search of reputable bibliographic databases, indexing services (and platforms), and other supplementary sources, including Google Scholar and hand-searching^25^. The primary searches, both electronic and hand-searching, was performed simultaneously by the first author with the assistance of a professional university librarian from 15 May to 28 June 2019.

Before conducting the primary searches, a comprehensive search strategy was co-developed by the first author, subject specialist and professional university librarian, and reviewed by all authors to ensure the correct use of indexing terminology, and Medical Subject Headings (MeSH) descriptors. The search strategy was pilot tested on a subset of records from the PubMed database. It was then tailored to the syntax and subject headings of all other consulted databases. Details of the search strategies descriptors and truncation used, and the number of returned records are presented as supplementary material (Supplementary Appendix No.1 – Table 1).

*Electronic searches*—To identify relevant studies assessing the associations between food insecurity and metabolic risk factors, advanced search strategies were applied in the following electronic bibliographic databases (and platforms): PubMed, EBSCOhost (CINAHL with full text, Health Source - Nursing, MedLine), Ovid (Journals@Ovid Full Text), Web of Science (SCiELO Citation Index) and supplementary sources in Google Scholar platform. Furthermore, the first author browsed through the link entitled ‘Related Articles’ option, which searches for similar citations using an intricate algorithm that scans titles, abstracts and MeSH terms to detect more studies. No language restrictions were imposed to minimise the risk that eligible studies could be inadvertently excluded.

However, the year of publication was limited to between January 2015 and October 2019 to ensure that the evidence of associations is demonstrably rooted in the most recent and up-to-date scientific literature. This decision was chiefly because practitioners and decision-makers are somewhat interested and encouraged to make use of the most current evidence to inform policy debates^26^.

*Hand-search*—The electronic searches were further supplemented by hand-searching the bibliographies (reference lists) of all eligible studies for inclusion and the previously published systematic reviews of a relevant topic. This was done in order to identify very recent studies that have not yet been included and indexed by electronic databases or including studies from journals that are not indexed by electronic databases, and thus not captured by our comprehensive search strategies. In addition, several subject specialists, medical professionals, and university librarian experts in the field were approached to identify additional relevant studies.

##### (b) Search management

The records of retrieved articles through electronic databases and hand-searches that met the inclusion criteria were exported to EndNote X9 (version 19.1.0.12691), a reference manager software virtual library (Thomson Reuters, Stamford, CT, USA). The virtual library was created specifically for this study; for removing duplicates of the same records, screening, and study selection.

#### Step 4: Study selection

**Figure 1:**
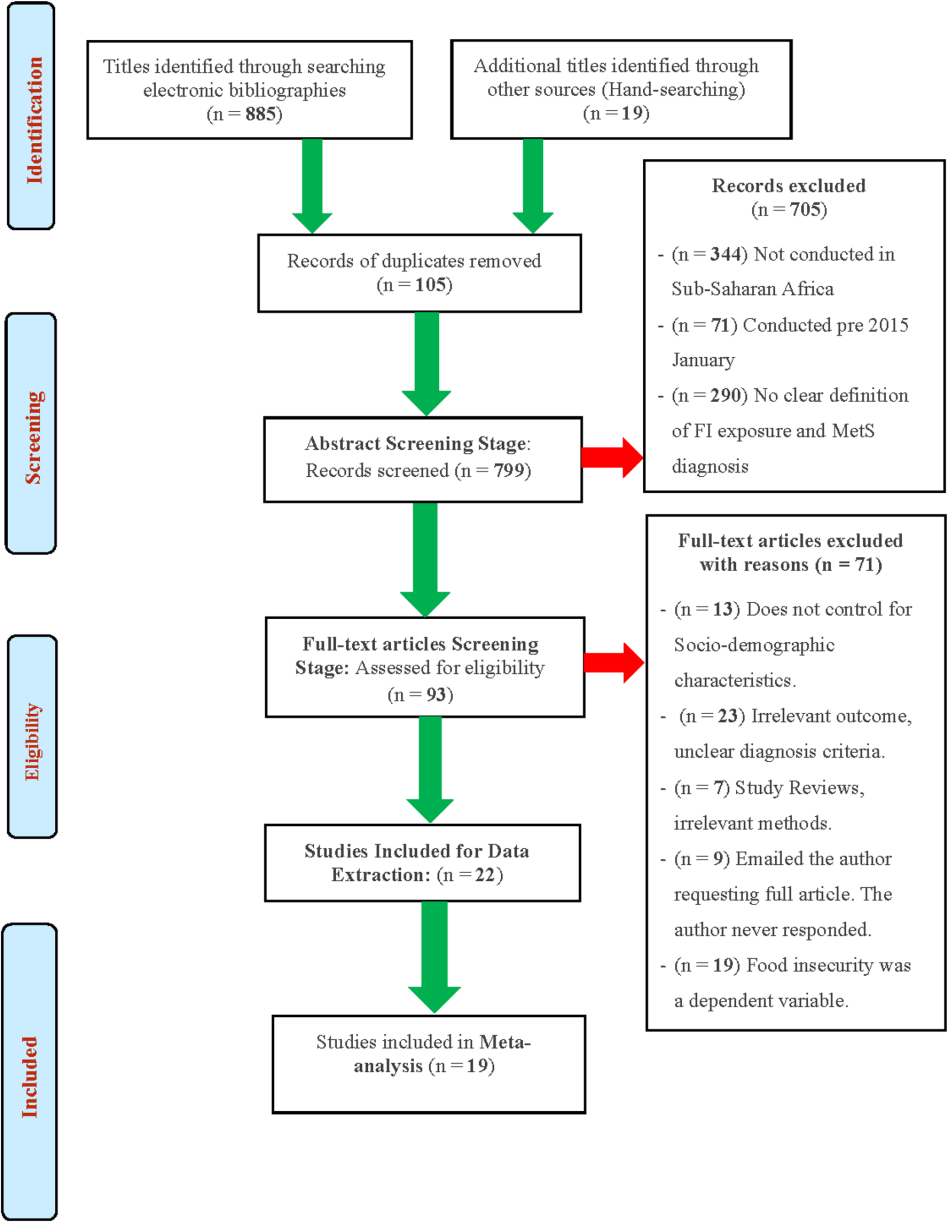
Preferred Reporting Items for Systematic Reviews and Meta-Analysis (PRISMA) flow diagram of literature search and study selection process. *Source*: Adopted from Ref.^20^

Study selection was a multi-step process that involved two reviewers. The procedure for screening of articles for eligibility was carried out in compliance with the *Preferred Reporting Items for Systematic Reviews and Meta-Analysis* (PRISMA) guidelines and summarised as a flow diagram Fig. 1^20^. The selection procedure involved three screening stages:

*Title screening*—Firstly, the first author with the assistance of a professional university librarian, searched for eligible titles by analysing the titles of articles found through the use of the search strategies, in the above mentioned electronic bibliographic databases (and platforms) guided by the study eligibility criteria (see Table 2). Articles with relevant titles were exported into a reference manager software – EndNote X9 (version 19.1.0.12691) virtual library. Duplicates were removed before the start of the abstract screening stage.

*Abstract screening*—And, subsequently, after the selection of relevant articles through the titles screening. Two skilled reviewers independently screened all the abstracts of the retrieved articles against the eligibility criteria (Table 2), utilising a standardised and pilot tested abstract screening checklist tool (see Supplementary Appendix No. 2 – Abstract Screening Google Form) for reliability and applicability. Differences between reviewers at this stage were settled through reaching a consensus among reviewers.

*Full-article screening*—Full-text articles were retrieved where studies met the inclusion criteria or where there was ambiguity to be screened in greater depth, by reference to the full-text assessment of eligibility. Thus, further establishing the retrieved studies’ eligibility, this stage was conducted like the abstract screening stage. Two independent reviewers utilised a standardised and pilot tested full-text screening checklist tool (see Supplementary Appendix No. 2 – Full-text Screening Google Form) for reliability and applicability.

At this stage, the disagreement was assessed using Cohen’s Kappa coefficient (κ) statistic on Stata 13.0SE (StataCorp College Station, TX, USA), a robust statistic used for inter-rater reliability testing (Supplementary Appendix No. 1 – Table 5)^27^. Detected differences between reviewers were resolved through discussion and a third arbitrator.

The excluded articles at each screening stage were stored in a different EndNote X9 virtual library. Reasons for exclusion of studies were documented per each stage (see Fig. 1).

#### Step 5: Data extraction

The data extraction procedure required two professional reviewers who extracted data separately and in duplicate to detect inter-rater errors and reduce data errors and bias. A standardised data extraction tool prepared in a Google Form format, based on a checklist provided in the *Cochrane Handbook for Systemic Reviews for Intervention*, was used^28^. Feedback was solicited from all authors regarding the draft list of data variables for extraction. The final data extraction tool was calibrated and piloted for reliability and applicability on the first three eligible studies to maintain accuracy and ensure all necessary information is captured.

For every study that met our eligibility criteria for inclusion, two reviewers working independently and in duplicate extracted essential data related to study characteristics, assessment of food insecurity, metabolic risk diagnoses, crude prevalence estimates (number of cases divided by the sample size) and outcome of interest measures (effect size data including the prevalence of key metabolic risk factors, measures of prospective associations with 95% confidence intervals, and covariates evaluated). A full list of data items extracted from each included studies is presented as supplementary material (Supplementary Appendix No. 2 – Data Extraction Google Form). The coordinator resolved disagreements between the two reviewers, and the decision was either taken to have a third reviewer to re-examine the study or extract all the necessary data from the studies concerned, guided by the same extraction process as outlined above.

In this meta-analysis, we extracted and used measures of associations that were the most adjusted for one or more sets of potential confounders such as socio-demographic and lifestyle factors only to reduce confounding and measurement errors, ensure consistency across studies and reduce bias. For more detail, missing details or inconsistencies, the authors of the included studies were contacted by e-mail, if any, with up to three attempts. The studies concerned were excluded after three unsuccessful attempts to contact the author.

#### Step 6: Assessment of study quality and risk of bias

The methodological quality (or risk of bias) of each included study was assessed by two reviewers independently, using the *Mixed Methods Appraisal Tool* (MMAT) – Version 2018^21^. The appraisal tool consists of two core assessment domains addressing the external validity (risk of selection, data collection and non-response bias) and five further assessment domains addressing the internal validity (systematic error—the risk of measurement bias and bias related to data analysis) deemed relevant to appraise the methodological quality of cross-sectional studies^22^. The summary of the domains critically assessed is provided as supplementary material (Supplementary Appendix No. 2 file).

In order to generate an overall risk of bias assessment, an overall percentage quality score was calculated for each included study (study quality of reporting) based on the assessment of each domain and the overall risk of bias across domains. The scores were interpreted as of low quality if ≤ 50%—average quality if 51–75%—and high quality if 76–100%. Differences in rating quality scores were resolved by discussion among the authors until consensus was reached. However, studies were not excluded based on quality, but quality scores were reported descriptively by indicating the methodological issues in each study and how these may influence the interpretation of the overall quality of evidence for each outcome.

The overall quality of evidence (or certainty in the findings) for each outcome collected was assessed based on study methodological quality, results from sensitivity analysis, and by downgrading and upgrading the baseline quality score for cross-sectional studies, according to the domains specified in the *grades of recommendation, assessment, development, and evaluation* (GRADE) guidelines^29^. The completed GRADE checklist and reasons to up-or down-grade quality of evidence in GRADE is provided as supplementary material (Supplementary Appendix No. 1 – Table 4).

#### Step 7: Data synthesis and analysis of results

##### (a) The primary outcome of interest—evidence of the associations between food insecurity and key metabolic risk factors

The primary outcome of interest in the current systematic review was evidence of associations between food insecurity and key metabolic risk factors for diet-sensitive NCDs, as reported in studies conducted in sub-Saharan African populations. However, upon re-evaluating the included studies and the relevant data extracted from these studies, we recognised the need to stratify the evidence of associations as reported by the studies into three strata of associations: Adverse Association, Non-significant Association, and Inconclusive Association.

1. [−] Adverse Association—Food insecurity was reported to be significantly associated with ≥ one key metabolic risk factor (observed with relevant statistical tool);
2. [+] Non-significant Association—There was no significant association found between food insecurity and reported key metabolic risk factors (observed with relevant statistical tool); and
3. [~] Inconclusive Association—The association between food insecurity and reported metabolic risk outcomes were inconclusive because, although exposure to food insecurity showed a protective effect toward reported metabolic risk factors, it was not statistically significant (observed with relevant statistical tool).

**Figure 2:**
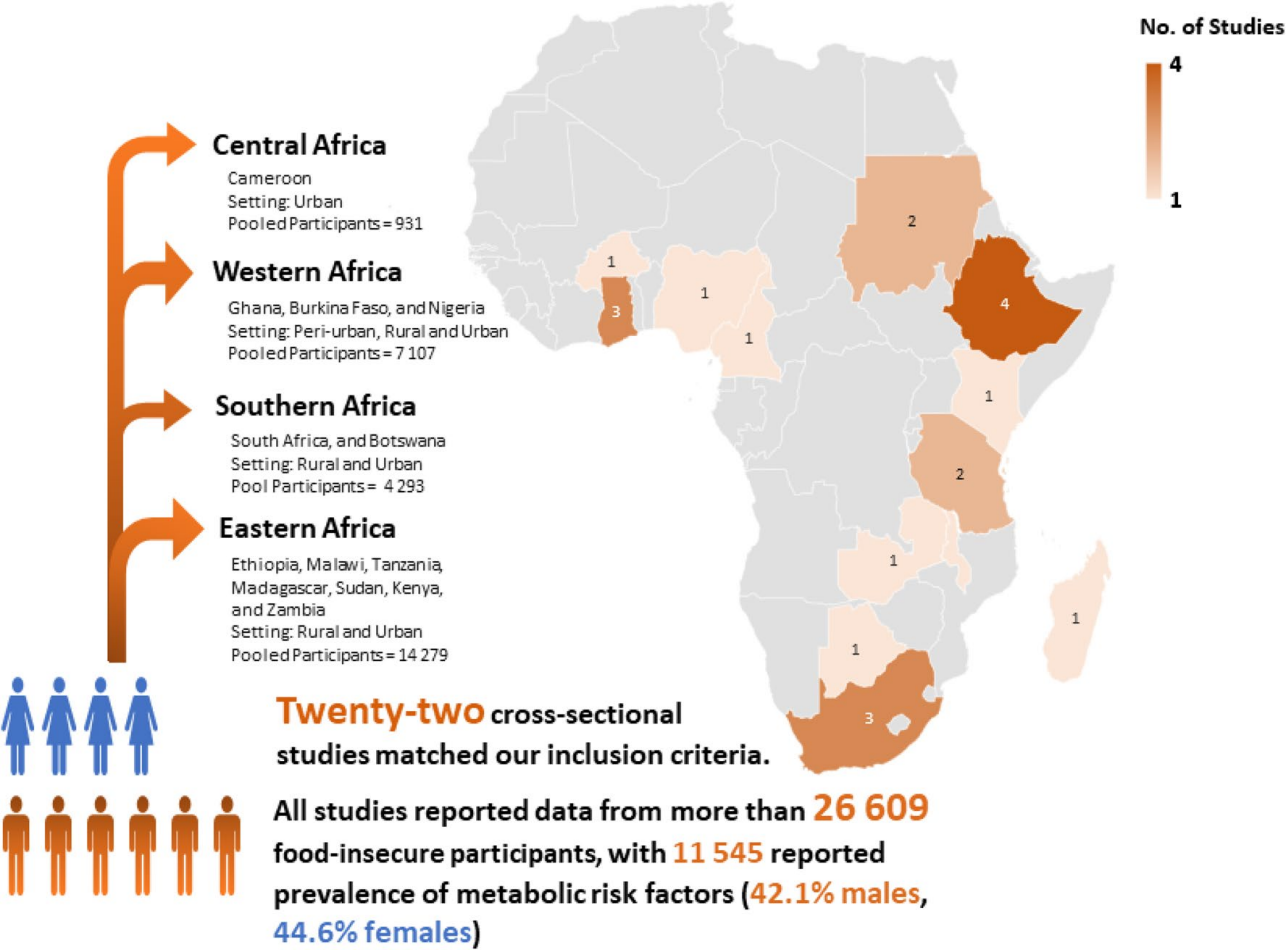
Map of Africa indicating the regions where the included studies were situated and the number of pooled study participants [Figure generated by the first author using Canva version 2.93.0^30^, Available from: http://about.canva.com/].

Furthermore, the reporting of the association effects relative to food insecurity was inconsistent across included studies (illustrated in Table 3); thus a narrative synthesis was performed to identify the specific key metabolic risk factors associated with food insecurity in sub-Saharan Africa. The included studies were manually coded into strata’ as explained above, and presented in the compiled summary of the extracted data table (see Table 3). The authors then used Canva version 2.93.0, an interactive web-based graphic design tool, to model an African map showing the four regions where the included studies and the number of study participants were located (Fig. 2)^30^.

**Table 3:** Characteristics of 22 included studies marching eligibility criteria, reporting the association between food insecurity and key metabolic risk factors in sub-Saharan Africa.

Author, publication year	Country, geographical setting	Population details, age (y)	Sample size, gender		Method of ascertainment		Significant findings		
					Food insecurity	Criterion for diagnosis	The outcome, n diagnosed*		Reported association effects relative to FI
Abebe, 2017^49^	Ethiopia, rural and urban	All permanent residents of the HDSS site, ≥ 1 year	1160	M—35.3% F—4.7%	Food insecurity access scale	Self-reported—‘Yes’ when asked whether they ‘ever had clinician diagnosed HTN?’	Hypertension	M—137 F—261	[−] Household food insecurity was significantly associated with hypertension (AOR = 1.71; 95% CI 1.37 to 2.12)
Agaba, 2017^33^	Nigeria, Urban	All employees of the university, ≥ 18 years	883	M—59.0% F—41.0%	Minimum Adequate Diet	The WHO STEPwise approach to chronic disease risk factor surveillance	Dyslipidaemia	M—254 F—199	[−] Inadequate intake of fruit and vegetables, was significantly associated with the reported Diet-Sensitive NCDs Risk Factor (94.6%; 95% CI: 92.8–95.9)
Anteneh, 2015^34^	Ethiopia, urban	School adolescents, 10—24 years	431	M—41.1% F—58.9%	Minimum adequate diet	The WHO STEPwise approach to chronic disease risk factor surveillance	Obesity	M—22 F—50	[−] Inadequate diet significantly associated with Obesity: 19.83, 95% CI: 3.96–99.23)
Colecraft, 2018^48^	Ghana, Semi-Rural	Residents of asesewa in the upper manya krobo district, ≥ 25 years	1165	M—44.0% F—56.0%	Minimum dietary diversity	The physical examination to confirm hypertension diagnosis completed by a nurse	Hypertension	M—28 F—35	[−] Below Minimum Dietary Diversity was found to be a contributory factor to having either an isolated elevated SBP or both elevated SBP and DBP
Cox, 2016 ^43^	Malawi, Rural	Children in rural Monkey Bay, ≤ 5 years	828	M—62.2% F—37.8%	Weight-for height is used as an indicator of nutritional status	Integrated management of childhood Illness (IMCI) guidelines by doctors	Acute respiratory infections (ARI)	M—45 F—24	[-] Acute-on-chronic malnutrition (OR 1.98, 95% CI 1.12–3.50, P = .02) and acute malnutrition (OR 2.62, 95% CI 1.17–5.89, P = .02) was significantly associated with having ARI
Desalew, 2017^36^	Ethiopia, Urban	Primary school children, 11—15 years	448	M—41.7% F—58.3%	Minimum Adequate Diet	The WHO Obesity and Overweight fact sheet	Obesity, Overweight and Underweight	M—61 F—85	[−] A significant association between Low fruit/vegetable intake per day and reported metabolic risk-AOR 1.3 (0.3, 5.1); 1.2 (0.3,5.4)
Di Gioia, 2016 ^35^	Madagascar, Urban	Enrolled children who came for routine visits, 4–6 years	313	M—47% F—53%	Malnutrition status according to BMI/ Quetelet index	An echocardiographic evaluation performed by a doctor	left ventricular mass	M—63 F—61	[−] LVMI values increased in proportion to BMI percentiles 29.8 ± 10.8 g/m 2.7 in the malnutrition group
Gebremichael, 2019^50^	Ethiopia, rural and urban	Hypertensive patients in a specialized hospital, ≥ 18 years	320	M—48.8% F—51.2%	Low level of adherence to DASH—A score of 6 or better	Self-care practice adopted from hypertension self-care activity level effects (H-scale)	Hypertension	M—92 F—76	[ +] No significant association Non-Adherence to dietary management 0.949 (0.508–1.772)
Soubeiga, 2017^51^	Burkina Faso, rural and urban	Nationally Representative Sample, 25 to 64 years	4,629	M—48.2% F—51.8%	Minimum Adequate Diet	The WHO STEPwise approach to chronic disease risk factor surveillance	Hypertension, Obesity, and Underweight	M—1114 F—1181	[-] A significant association between intake of butter versus none or vegetable oil (1.98, 1.22 to 3.22)
Katalambula, 2018^37^	Tanzania, Urban	Retrospective chart audit study in two national hospitals, 26–65 years	1450	M—47.2% F—52.8%	Minimum Dietary Diversity	Patient charts indicating a diagnosis of CRC per the ICD-10—WHO Version for 2016	Hypertension, Obesity, and overweight	M—239 F—276	[−] Healthy dietary pattern was significantly negatively correlated with HTN (ARR = 0.82, CI: 0.68–0.99)
Kejo, 2018^44^	Tanzania, Rural	Residents of Arusha District, ≤ 5 years	436	M—32.2% F—23.2%	Self-reported ‘yes’ to the practice of exclusive breastfeeding	Anthropometric data were collected through the measurement of length/height and weight of all children	Underweight, and Stunting	M—211 F—130	[−] None exclusively breastfed children were associated with underweight (AOR: 2.5, 95% CI: 1.0–6.3)
Lapauw, 2016^38^	Ghana, Urban	Female Residents of 3 slums in Accra, 15 – 49 years	250	M—0% F—100%	Minimum Dietary Diversity	The WHO STEPwise approach to chronic disease risk factor surveillance	Hypertension and Obesity	M—0 F—162	[−] Urban slum women who had middle dietary diversity had greater odds (OR, 1.13; CI, 0.66, 1.92) of developing MetS
Maimela, 2016^45^	South Africa, Rural	Permanent residents of the Dikgale HDSS site, ≥ 15 years	1403	M—37.4% F—62.6%	Minimum Adequate Diet	The WHO STEPwise approach to chronic disease risk factor surveillance	Hypertension, Obesity, and overweight	M—337 F—835	[−] People who had low fruit and vegetable intake were found to be 1.8 times more likely to have MetS (*p* < 0.05)
Mbaissouroum, 2017^52^	South Africa, Rural and Urban	South African Citizens, ≥ 60 years	2145	M—41% F—59%	Minimum Adequate Diet	The WHO Study on Global Aging and Adult Health (SAGE)	Hypertension	M—225 F—569	[−] Inadequate intake of fruit and vegetables, was significantly associated with the reported HTN
Mohammed, 2016^47^	Ghana, Peri-urban	Permanent residents in two peri-urban communities, 18–45 years	180	M—50% F—50%	Minimum Dietary Diversity	Self-reported, medical records and physical examination	Hypertension, Obesity, Overweight and Underweight	M—52 F—72	[−] Increased dietary diversity score was associated with decreased MetS prevalence (*p* <0 .05)
Musaiger, 2016^39^	Sudan, Urban	University students in the College of Education, 18 to 30 years	400	M—45.8% F—54.3%	Minimum Meal Frequency, adequate diet	Self-reported and Anthropometric measurement	Obesity, Overweight and Underweight	M—58 F—88	[−] Inadequate intake of fruit, vegetables, fast food significantly associated with reported MetS
Musaiger, 2016 ^40^	Sudan, Urban	Schoolchildren in public schools aged 14 to 18 years	945	M—53.7% F—46.4%	Minimum Adequate Diet	The International Obesity Task Force standard	Obesity and Overweight	M—46 F—54	[−] Unhealthy dietary habits were significantly associated with the reported MetS
Mutisya, 2015^41^	Kenya, Urban	All women of reproductive health who are residents, ≤ 5 years	6858	M—53.7% F—43.9%	Food Insecurity Access Scale	Z-Scores for the HFA using the ‘WHO Child Growth Charts	Stunting and Underweight	M—1858 F—1491	[−] risk of stunting increased significantly by 19 and 22% among children from moderately and severely food insecure households
Solomons, 2018^53^	South Africa, Rural and Urban	All residents of Langa, the urban PURE study site in the Western Cape province, 32 to 81 year	454	M—24.9% F—75.1%	Minimum Adequate Diet, Low adherence to DASH	Self-care practice adopted from hypertension self-care activity level effects (H-scale)	Hypertension	M—60 F—172	[−] Significant negative correlations were found between dietary adherence score (r = − 0.108)
Omech, 2016^54^	Botswana, Rural and Urban	Outpatients aged ≥ 20 years without a diagnosis of diabetes mellitus	291	M—25.8% F—74.2%	Minimum Dietary Diversity, Adequate Diet	Metabolic syndrome was assessed using the National Cholesterol Education Program-Adult Treatment Panel	Dyslipidaemia and Obesity	M—34 F—129	[~] Although consumption of vegetables, fruit, and berries showed a protective effect toward MetS, it was not statistically significant
Nansseu, 2019^42^	Cameroon, Urban	Students aged 18–35 years, with no known history of CVD	931	M—53.8% F -46.2%	Minimum Adequate Diet	Validated questionnaires, Anthropometric indicators, and Biochemical analyses, including fasting blood samples	Hypertension, Obesity, Overweight and Underweight	M—134 F—173	[-] Inadequate intake of fruit and vegetables was significantly associated with the reported MetS (99.0%; 95% CI 98.4 to 99.6)
Tateyama, 2018 ^46^	Zambia, Rural	Residents of the Mumbwa district aged 25–64 years	690	M—48.6% F—51.4%	Minimum Adequate Diet, Food Insecurity access scale	The WHO STEPwise approach to chronic disease risk factor surveillance	Dyslipidaemia Hypertension, and Obesity	M—169 F—183	[−] Inadequate intake of fruit and vegetables (AOR, 1.97; 95% CI, 1.05–3.68) were significantly associated with BMI 25 kg/m^2^

[−] Adverse—Food insecurity was reported to be significantly associated with ≥ one critical metabolic risk factors.

[+] Non-significant—There was no significant association between food insecurity and reported key metabolic risk factors.

[~] Inconclusive—The association between food insecurity and reported metabolic risk factors was inconclusive because, although exposure to food insecurity showed a protective effect toward MetS, it was not statistically significant.

*n diagnosed—the number of participants diagnosed with the reported metabolic risk factor, for male (M) and females (F).

##### (b) The secondary outcome of interest—prevalence estimates of metabolic risk factors associated with food insecurity among the food-insecure patients

After analysing the primary outcome, and identifying the spectrum of key metabolic risk factors associated with food insecurity, the secondary outcome was the prevalence of these metabolic risk factors among the food-insecure population in sub-Saharan Africa; and to visualise the variation patterns in the occurrence of these metabolic risk factors by gender and geographic area. A similar type of metabolic risk factors were pooled from each study for overall estimates.

##### (c) Statistical analysis

The prevalence estimates of key metabolic risk factors associated with food insecurity among the food-insecure patients were calculated by meta-analysis. The meta-analysis was performed using the statistical software MetaXL version 5.3 (EpiGear International, Sunrise Beach, Queenland, Australia) to combine all studies’ prevalence^31^. The pooled prevalence of metabolic risk factors and it’s 95% confidence interval (CI) were analysed using the random-effects model, utilising the inverse variance method of the DerSimonian-Laird approach to estimate the heterogeneity between-study population^31^. Forest plots were drawn showing the variation of the prevalence among all included studies together with the pooled prevalence estimates. Studies with a low risk of bias and outcomes deemed to be sufficiently homogenous; were selected for inclusion in the meta-analysis to compute the pooled prevalence estimates.

Heterogeneity in meta-analysis is inevitable due to differences in study quality, sample size, method, and outcome measurements across studies. Therefore, all meta-analyses were statistically, appraised for significant inter-study heterogeneity by using the I^2^ statistics for statistical significance of heterogeneity (i.e., the percentage of variance not due to studies-wide sampling error), where the significance was considered as per the guidelines of the *Cochrane Handbook for Systematic Reviews of Interventions*^28^. Consequently, an I^2^ value of ≥ 75% suggests a substantial heterogeneity.

Furthermore, by sequential omission of one study at a time, sensitivity analyses were performed to assess the meta-analysis results’ stability and evaluate the potential influence of single studies on the pooled prevalence estimates. Subgroup analyses were performed to evaluate the influence of the difference in age, gender, publication year, and geographic area among the included studies. Moreover, publication bias (Meta-biases) was assessed by visual inspection of the funnel plots (a plot of effect estimates against sample sizes) for asymmetry, complemented with the Egger regression test^23^. The graph’s symmetrical shape was interpreted as the absence of publication bias, while an asymmetrical shape of the graph was interpreted as the possibility of publication bias^28,32^. However, these quantitative tests were not run for meta-analyses with a small number of studies (n < 10).

##### (d) Principal summary measures

The included studies were cross-sectional study designs, the data for the relationship between food insecurity and key metabolic risk factors were measured and analysed cross-sectionally rather than longitudinally; therefore, the chief summary measures included prevalence along with percentages (%), 95% confidence intervals (95% CI) were reported. The results of this systematic review and meta-analysis were reported based on the Preferred Reporting Items for Systematic Review and Meta-Analysis statement (PRISMA) guidelines. The entire study screening, selection, and inclusion process are shown with a flow diagram’s support. Moreover, tables and narrative summaries are used to report the evidence of the associations between food insecurity and key metabolic risk factors and the risk of bias for every eligible study.

### Ethical approval and consent to participate

This paper is a Systematic Review and Meta-analysis study that relied strictly on the review of existing literature, and no human participants were involved. Therefore, ethical approval and consent to participate by human participants were not applicable.

## Results

### Screening results

The initial electronic bibliographic database searches yielded 11 803 potentially eligible articles (Supplementary Appendix No. 1 – Table 1). Following title screening, 10 918 articles were excluded, and 885 met the eligibility criteria and were exported to the EndNote X9. Hand-searching yielded 19 articles that could not be captured by the primary search strategies, which were also exported to EndNote X9 virtual library. A total of 105 duplicates was removed from the library, leaving 799 articles eligible for abstract screening. Following abstract screening, 93 articles were considered suitable for thorough assessment in the full-article screening stage, during which 71 of them were excluded. Full-article screening yielded 22, which were selected for inclusion in data extraction and were included in the narrative synthesis after passing the methodological quality assessment. Nineteen articles were deemed to be sufficiently homogenous for inclusion in the meta-analysis. See Fig. 1 for the PRISMA flow chart detailing the study selection and exclusion process.

The inter-rater reliability score for the full-article screening stage showed that there was a 92.5% agreement versus 62.2% expected by chance, which constitutes a considerably high agreement between screeners (Kappa statistic =  − 0.80 and *p*-value < 0.05) (see Supplementary Appendix No. 1 – Table 5). McNemar’s chi-square statistic suggests no statistically significant difference in the proportions of yes/no answers by reviewers with *p*-value > 0.05.

### Methodological quality (or risk of bias) assessment

The quality of the studies has been examined; Table 2 in the supplementary material (Supplementary Appendix No. 1 – Table 2 and 3) provides details of study quality based on the Mixed Methods Appraisal Tool (MMAT) – Version 2018^21^. In more recent studies, the methodological quality was generally higher, with one study rated the lowest (57.1%). The overall risk of bias across domains was judged to be low or unclear. We identified a potential source of bias in the confounding assessment; eight studies were found to have a high risk of bias. Overall, the GRADE quality of evidence for each outcome was assessed as very low quality of evidence to support these associations.

### Characteristics of included studies

Table 3 presents the baseline characteristics, and significant findings of all the included 22 studies with a total sample of 26,609 (46.8% males) food-insecure participants, with 11,545 (42.1% males) reported prevalence of metabolic risk factors (Table 4). The studies were conducted between 2015 and 2019, contributing very recent evidence of the associations between food insecurity and specific key metabolic risk factors observed among children (00–14 years), youths (15–24 years), and adults (25–64 years), and senior (≥ 65 years) participants (see Table 3).

**Table 4:** A qualitative description of key metabolic risk factors prevalence derived from 22 individual studies.

Studies reference	Reported type of metabolic risk factors	Participants diagnosed with metabolic risk factors	
		Male (5239)	Female (6306)
(n = 5 studies of high quality, n = 7 of average quality) ^34,^^36,^^40–^^42,^^45–^^47,^^51,^^54^	Obesity	326 (6.2%)	896 (14.2%)
(n = 2 studies of high quality, n = 1 of average quality^33,46,54^	Dyslipidemia	395 (7.5%)	235 (3.7%)
(n = 6 studies of high quality, n = 6 of average quality)^37,^^38,^^42,^^45–^^53^	Hypertension	1509 (28.8%)	2681 (42.5%)
(n = 2 studies of high quality, n = 4 of average quality)^36,^^39,^^42,^^44,^^47,^^51^	Underweight	592 (11.3%)	220 (3.5%)
(n = 2 studies of high quality, n = 5 of average quality)^36,^^37,^^39,^^40,^^42,^^45,^^47^	Overweight	370 (7.1%)	561 (8.9%)
(n = 2 of average quality)^41,44^	Stunting	1991 (38.0%)	1576 (25.0%)
(n = 1 of high quality)^43^	Acute Respiratory Infections (ARI)	45 (0.9%)	24 (0.4%)
(n = 1 of average quality)^35^	left ventricular mass (LVM)	51 (1.1%)	73 (1.2%)

All twenty-two included studies employed cross-sectional study design in stock and covered different geographic settings, distributed across four sub-Saharan Africa regions. A large proportion of the studies (n = 10 studies) was conducted in urban settings^33–42^, and four conducted in rural settings^43–46^. One study was conducted in a peri-urban^47^, and one study in a semi-rural setting^48^. The remaining six studies recruited participants from both rural and urban settings^49–54^. Figure 2 shows the distribution of the rest of the studies by country and regions of sub-Saharan Africa, with only 1 study from Central Africa^42^ and 12 from Eastern Africa.

#### (a) Food insecurity ascertainment

The adjustment for confounding factors was made in all twenty-two included studies; the most commonly adjusted factors were related to socio-demographic and lifestyle factors. The methods of assessing food insecurity exposure and ascertaining metabolic risk outcomes varied across the twenty-two included studies. Food insecurity exposure was generally measured either at a household level or at an individual level and indicated categorically.

In this group of studies, a subset (3 studies) involved designs where researchers measured food insecurity at the household level using the Household Food Insecurity Access Scale (a six-item short questionnaire), a valid scale for assessing food insecurity^41,46,49^. If the subjects were responded to two or more of the six items, they were classified as food insecure. Another subset (9 studies) measured food insecurity at an individual level using the Minimum Adequate Diet criterion^33,34,36,40,42,45,46,51,52^, and the Minimum Dietary Diversity criterion (5 studies)^37,38,47,48,54^, in which the individuals confronted with food insecurity had reported lower rates of consumption of fruits, vegetables, and lower proportion of eating from less than four food groups, due to inconsistent access to food.

The last subset (4 studies) used more than one tool to gauge food insecurity^39,46,53,54^. While studies on children ≤ 5 years used the Minimum Meal Frequency criterion^39^, the Weight-for-height criterion^43^, and the Quetelet index^35^. However, of the studies that examined the age ranges linked to childhood and adolescence, none considered the findings separately by groups of children and teenagers since they vary in terms of growth, progress, and maturation. These variations can impact the existence of metabolic risk factors. See Supplementary Appendix No. 1 – Table 6 for a detailed list of reported tools/ methods definitions and theoretical minimum risk exposure level/ scales used to ascertain food insecurity for each study.

#### (b) Metabolic risk factors ascertainment

Collectively, the included studies assessed the association between food insecurity and a variety of specific key metabolic risk factors, using a total of ten international diagnostic criteria to diagnose metabolic risk outcomes (see Supplementary Appendix No. 1 – Table 7). The WHO STEPwise approach to chronic disease risk factor surveillance diagnostic criteria was found to be the most used diagnostic tool (6 studies)^33,34,36–38,45,46,51^; while a combination of a self-reported pretested questionnaire administered by a nurse or doctor followed by either an anthropometric measurement, screening of medical records, physical examination, and biochemical analyses to confirm condition—was the second most used diagnostic tool (5 studies)^39,42,47,49,50^. One study on seniors ≥ 60 years used the WHO Global Aging and Adult Health (SAGE) criteria to diagnose metabolic risk outcomes^52^. The rest of the included studies use either hypertension self-care activity level effects (H-scale), Integrated Management of Childhood Illness (IMCI) guidelines, the WHO Obesity and Overweight fact sheet guidelines, echocardiographic evaluation, patient charts indicating a diagnosis per the ICD-10—WHO Version for 2016, the International Obesity Task Force guidelines, height-for-age Z-scores for the HFA using the ‘WHO Child Growth Charts, and lastly The National Cholesterol Education Program-Adult Treatment Panel.

### Results of individual studies

The systematic review revealed evidence on the associations between food insecurity and specific key metabolic risk factors on the causal pathway to diet-sensitive non-communicable diseases (NCDs). These metabolic risk factors are dyslipidaemia, underweight, left ventricular mass, obesity, hypertension, acute respiratory infections (ARI), and overweight observed in children (00–14 years), youths (15–24 years), adults (25–64 years), and senior (≥ 65 years) participants, pooled across sub-Saharan African countries.

#### (a) The primary outcome—evidence of the associations between food insecurity and key metabolic risk factors

The associations between food insecurity and metabolic risk outcomes were identified by coding the accumulated evidence on the associations into three strata, namely: [−] Adverse associations, [+] Non-significant associations, and [~] Inconclusive associations. In summary, Table 3 details the significant findings of all the twenty-two included studies.

In strata 1, 20 of the 22 studies reported that exposure to food insecurity might be adversely associated with the presence of at least one analyzed metabolic risk factor. Of the 20 studies demonstrating a significant association, 14 were of high quality and 6 of average quality. The association existed even for marginal food security (a less severe level of household food insecurity) (Table 3)^33,34,37–40,42,45–49,51–53^.

Seven studies with children as participants (00–14 years) within this stratum reported an increased risk of stunting and underweight in children from moderately and severely food-insecure households by 19 and 22%^36,41,44^. The association between left ventricular mass (LVM) and malnutrition was found in Di Gioia et al.^35^, in which the population with food insecurity reported statistically lower LVM index values (29.3 ± 10.1 g/m^2,7^ vs. 33.6 ± 12.5 g/m^2,7^, *p* = 0.001), relative to normal nutritional status (29.8 ± 10.8 g/m^2,7^ vs. 32.9 ± 12.1 g/m^2,7^, *p* = 0.02). Cox et al.^43^ observed that acute-on-chronic malnutrition and acute malnutrition was significantly associated with having acute respiratory infections (ARI) which was demonstrated with AOR of 1.98 (95% CI: 1.12–3.50, *p* = 0.02) and 2.62 (95% CI: 1.17–5.89, *p* = 0.02) respectively.

However, in strata 2, Gebremichael et al.^50^ in adults and senior based study in Tigray, Ethiopia (n = 1 of high quality, 100%), showed a statistically non-significant AOR of 0.949 (95% CI: 0.508–1.772, *p* = 0.869) demonstrating a slight positive association between hypertension and exposure to below minimum adequate diet, in which the population with food insecurity reported fewer than five servings of fruits and vegetables per day.

Finally, in strata 3, Omech et al.^54^ assessed the associations between dyslipidaemia, obesity and exposure to food insecurity in an adults-only study participant (n = 1 of average quality, 71%). He found that although a low rate of daily consumption of vegetables, fruit, and berries showed a protective effect on dyslipidaemia and obesity, it was not statistically significant and was not included in the multivariate analysis. Thus, the association between dyslipidemia, obesity, and exposure to food insecurity was inconclusive. More studies are warranted on stratum two and three reported associations; as one study is insufficient and possible reasons for this are discussed under the limitation’s section.

#### (b) The secondary outcome—prevalence estimates of metabolic risk factors associated with food insecurity among the food-insecure patients

**Figure 3:**
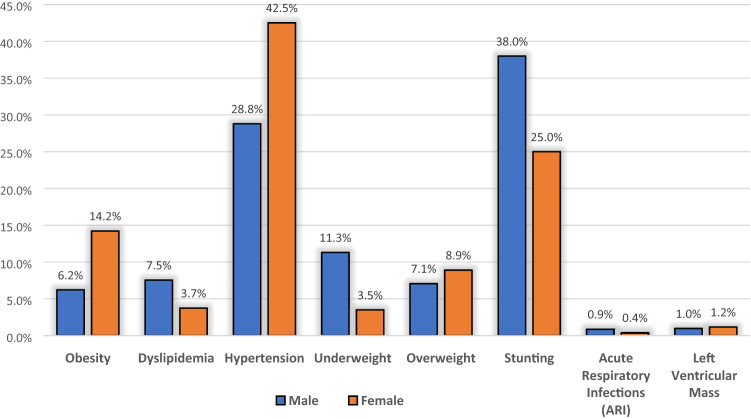
The metabolic risk factors prevalence derived from 22 studies by gender strata.

After identifying the spectrum of key metabolic risk factors associated with food insecurity, we conducted a meta-analysis to determine the pooled prevalence estimates of this spectrum of metabolic risk factors among the food-insecure patients in sub-Saharan Africa. The prevalence estimates of these metabolic risk factors pooled from the 22 included studies, differed significantly across gender, as Fig. 3 and Table 4 illustrates. This analysis was done to identify the population groups most likely at higher risk by visualising the prevalence profile of metabolic risk factors; predisposing them to develop diet-sensitive NCDs such as diabetes or cardiovascular diseases.

The twenty-two included studies collectively presented prevalence data on 26,609 (46.8% males) residents of sub-Saharan Africa. The frequency Table 4 shows the pooled number of participants diagnosed with the spectrum of metabolic risk factors relative to the total number diagnosed for males and females. The percentage with respects to these totals is shown in parenthesis. Notably, there appears to be a slight difference between female participants (44.6%) diagnosed with the different key metabolic risk factors and their male counterparts (42.1%) who were also diagnosed. Regarding geographic distribution, the reported prevalence of metabolic risk factors among the food-insecure patients in sub-Saharan Africa ranged from the lowest in Western Africa, Ghana (63/1165; 5.4%)^48^ to the highest in Southern Africa, South Africa (1172/1403; 83.5%)^45^ (Table 3: pooled estimate was found to be 41.8%, 95% CI: 33.2% to 50.8%, I^2^ = 99.5% *p*-value < 0.00).

### Results of meta-analysis

19 out of the 22 included studies were of sufficient quality for inclusion for meta-analysis (Fig. 1). A substantial degree of heterogeneity was detected across studies from the 19 studies assessing the associations between food insecurity and metabolic risk factors. Possible reasons for this are discussed under the limitation’s section.

**Figure 4:**
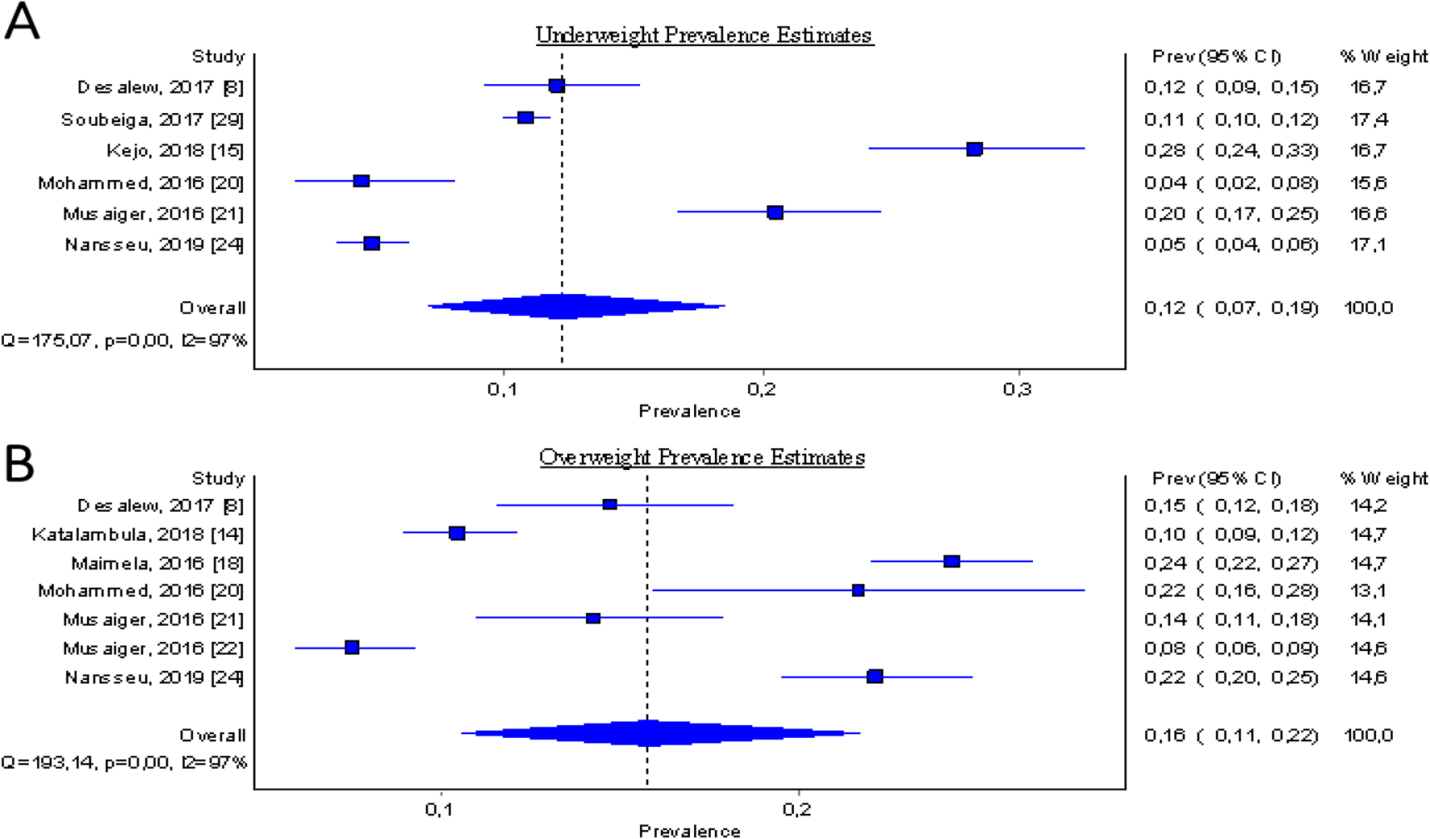
Forest plot showing study-specific and pooled estimates for being underweight and overweight prevalence in Sub-Saharan Africa, from studies published during the period 2015–2019, (**A**: The pooled prevalence estimates for being underweight; **B**: The pooled prevalence estimates for being overweight).

**Figure 5:**
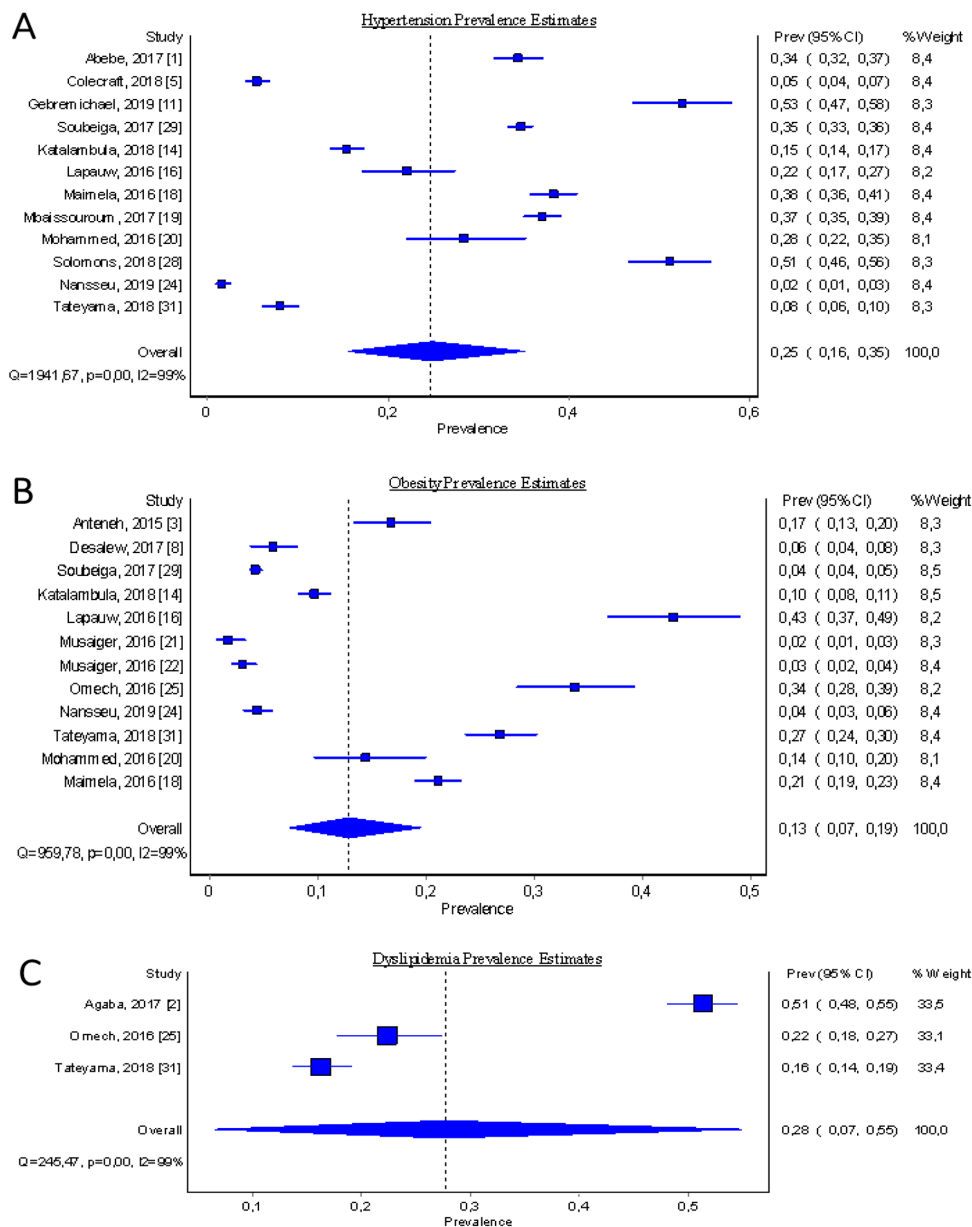
Forest plot showing study-specific and (**A**): The pooled prevalence estimates for hypertension; (**B**) The pooled prevalence estimates for obesity; (**C**) The pooled prevalence estimates for dyslipidaemia.

Concerning pooled prevalence estimates of specific key metabolic risk factors among the food-insecure patients, we tried to combine similar metabolic risk factors pooled from various studies with the number of participants diagnosed with that outcome factor. The results are summarised and presented in the forest plots (Figs. 4, 5). However, due to the low number of studies that reported stunting, acute respiratory infection (ARI), and left ventricular mass (LVM) types of metabolic risk factors (less than 3), a meta-analysis could not be undertaken for those studies.

#### (a) Pooled prevalence estimates for being the underweight and overweight among food-insecure participants

The pooled prevalence estimates for being underweight among the food insecure participants, derived from six studies, was found to be 12.2% (95% CI: 7.0% to 18.5%), irrespective of diagnostic criteria (Fig. 4A). Substantial heterogeneity was detected by I^2^ statistic between each of the six studies (I^2^ = 97.14% *p*-value < 0.00). The pooled prevalence estimates for being overweight among the food insecure participants, derived from seven studies, was found to be 15.8% (95% CI: 10.6% to 21.7%) and the observed heterogeneity detected at I^2^ = 96.89% *p*-value < 0.00 (Fig. 4B).

#### (b) Pooled prevalence estimates for hypertension, obesity, and dyslipidaemia among the food insecure participants

The pooled prevalence estimates for hypertension and obesity among the food insecure participants, derived from 12 studies was found to be 24.7% (95% CI: 15.6 to 35.1%, I^2^ = 99.4% *p*-value < 0.00) (Fig. 5A), and 12.8% (95% CI: 7.4 to 19.5%, I^2^ = 98.85% *p*-value < 0.00) respectively (Fig. 5B). The pooled prevalence estimates for dyslipidaemia among the food insecure participants, derived from 3 studies was found to be 27.6% (95% CI: 6.5% to 54.9%), heterogeneity was detected by I^2^ statistic at I^2^ = 99.18% *p*-value < 0.00 (Fig. 5C).

#### (c) Exploration of heterogeneity

**Figure 6:**
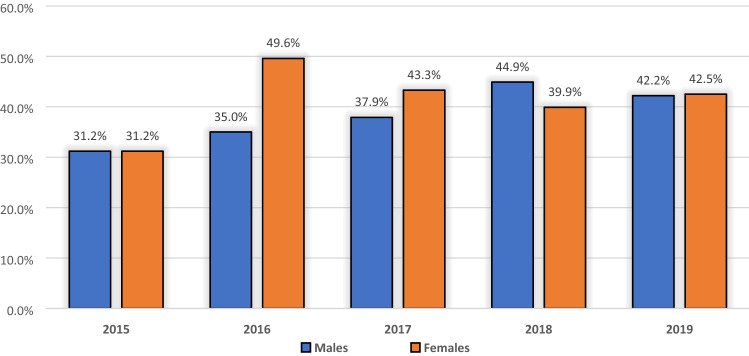
Pooled estimates for metabolic risk factors prevalence by year of publication.

Across the included studies in the meta-analyses, substantial heterogeneity was detected in all the pooled estimations. Due to the small number of studies (n < 10), thus providing limited ability to detect the cause of heterogeneity; the exploration of potential sources of significant heterogeneity was not formally conducted. Secondly, we could not explore publication bias with examining the funnel plots for asymmetry, for the reason that in the presence of high heterogeneity, there is no reason to expect a plot of estimates against their SEs to have a funnel shape. However, pooling of estimates according to the year of publication suggests an increase in metabolic risk factors prevalence over time: 31.6% (95% CI: 2.2% to 67.3%) across studies published in 2015; increasing to 44.7% (95% CI: 17.4% to 73.1%) from studies published after 2015. The variations between males and females are shown in Fig. 6.

#### (d) Sensitivity analyses

We explored the influence of each study on the overall meta-analyses’ summary estimates. Accordingly, there was no significant improvement on the degree of heterogeneity, compared to the combined estimate obtained by omitting any one single study included in these meta-analyses or any of the expected outliers.

## Discussion

Metabolic risk factors warrant particular public health effort, due to their substantial contribution to global disease burden, rapidly increasing trends, and variable patterns in incidence by age, gender, or region across countries^4,8,10^.

While there are progressively better estimates of the prevalence of metabolic risk factors and proximate drivers of this prevalence in high-income countries; reliable data on the trend and patterns of key metabolic risk factors among the food insecure population in resource-limited settings, such as sub-Saharan African countries still lag far behind^55^. The primary objective of this systematic review and meta-analysis was to use accumulated evidence published between January 2015 and June 2019, to determine the associations between food insecurity and key metabolic risk factors for diet-sensitive NCDs. After analysing the accumulated evidence of associations, and identifying the spectrum of metabolic risk factors associated with food insecurity, the secondary outcome was the prevalence estimates of these metabolic risk factors among the food insecure participants in sub-Saharan Africa.

Our systematic review findings revealed that exposure to food insecurity might be adversely associated with the presence of specific key metabolic risk factors on the causal pathway to diet-sensitive NCDs in children (00–14 years), youths (15–24 years), adults (25–64 years), and senior (≥ 65 years) participants. In the context of sub-Saharan Africa, food insecurity affects approximately more than 257 million (1 in 3) people^4^; our meta-analysis pooled prevalence estimate for metabolic risk factors, was found to be 41.8% (95% CI: 33.2 to 50.8%) among the food-insecure participants. This finding indicates that more than two individuals out of five (2 in 5) are diagnosed with at least one of the analysed metabolic risk outcomes, such as dyslipidaemia, underweight, left ventricular mass (LVM), obesity, hypertension, acute respiratory infections (ARI), and overweight on the causal pathway to diet-sensitive NCDs.

Thus, generally, the results of both analyses demonstrate that in sub-Saharan Africa, the food insecure population is disproportionately at higher risk of developing diet-sensitive NCDs, indicated by the significantly high pooled estimates for metabolic risk factors prevalence. Since this was the first meta-analysis (quantitative synthesis) regarding the epidemiology of metabolic risks among the food insecure population in sub-Saharan Africa; we tried to compare our findings to other surveys and reviews conducted outside of the sub-Saharan Africa or countries under study.

### Comparison with previous studies

Previous systematic reviews and several cross-sectional studies have contributed to the evidence that food insecurity is associated with metabolic risk factors on the causal pathway to diet-sensitive NCDs. In the current review, the results of both analyses show imperative similarities, consistent with and expands the results of earlier reports. In addition, the current review yields new evidence visualising variation patterns in the prevalence of specific key metabolic risk factors by gender and geographic area among the food insecure population in sub-Saharan Africa. Thus, providing subsidies for public health intervention; while affirming the need to tackle food insecurity in order to effectively promote population health and the prevention and control of diet-sensitive NCDs, among the food insecure population^56^.

Similar to our systematic review, Arenas et al.^5^ found an adverse association between exposure to food insecurity and dyslipidemia, as reported from 7 peer-reviewed manuscripts published between 2006 and 2015, involving only US participants. However, no meta-analysis was performed, and some of these studies used self-reported dyslipidemia. A recent review by Beltran et al.^57^ showed that most studies in the USA that indicate a correlation between food insecurity and hypertension correspond to self-reporting of conditions. They stated that a likely anxiety connection complicates the relationship between self-reported metabolic risk factors and food insecurity (since anxiety is also increased in food-insecure patients)^58^.

On the other hand, Arenas et al.^5^ found no consistent literature-wide association between body mass index and food insecurity-status; only 18 studies out of 37 reported a statistically significant association, which is more studies than we report in our current systematic review. While not all studies could confirm these associations, these findings have important implications for future research, as the inconsistencies of results across studies merit careful assessment of the various methodologies and harmonized classification criteria used in the original studies. In general, potential sources of inconsistencies across studies may be ascribed mostly to various methodological concerns, relating to the design of the studies, analytical techniques, the diversity of food insecurity and malnutrition indicators used, wide variations in clinical diagnostic criteria for defining metabolic risk factors and, above all, to the limited availability of high-quality micro-level data from large-scale surveys^59–61^.

Notwithstanding the inconsistencies of results across studies examining the associations between food insecurity and key metabolic risk factors; the random-effect meta-analytic component of the current review provides the opportunity to present estimates of the prevalence of the spectrum of metabolic risk factors associated with food insecurity after identifying these metabolic risk factors among the food insecure population in sub-Saharan Africa. Ahmed et al*.*^62^ in Saudi Arabia, estimated a comparable overall prevalence of metabolic risk factors of 41.8% from The Africa Middle East Cardiovascular Epidemiological (ACE) study cohort. Earlier community survey reports in other sub-Saharan African countries reported a lower prevalence among adult participants in Ethiopia, Kenya, and South Africa, ranging from 17.9% reported in 2011 to 34.6% reported in 2012, and 30.7% reported in 2014, respectively^63–65^. Our meta-analysis findings add to this evidence of a rapidly growing burden of metabolic risk factors between different populations, indicating a substantial increase in risk for diet-sensitive NCDs in the sub-Saharan Africa region over the years.

Moreover, visualising variation patterns in occurrence by gender among the pooled food-insecure participants, an almost twofold female-to-male disparity in the prevalence of key metabolic risk factors were observed. Our findings indicate that dyslipidaemia is the most prevalent type of metabolic risk factor among males (7.5%), but not among females (3.7%) derived from 3 studies with the pooled prevalence estimate of 27.6% (95% CI: 6.5% to 54.9%, I^2^ = 99.18% *p*-value < 0.00)^33,46,54^. Hypertension was the second most prevalent type of metabolic risk factor with an estimate of 24.7% (95% CI: 15.6% to 35.1%, I^2^ = 99.43% *p*-value < 0.00), higher among females (42.5%) compared to males (28.8%), followed by obesity (females 14.2%, males 6.2%), and lastly, being overweight (females 8.9%, males 7.1%). These findings have implications for accurate prioritisation of food insecurity and metabolic risk factors screening for effective utilisation of insufficient health services in resource-limited environments, including sub-Saharan Africa.

Moreover, these findings are consistent with Motala et al.^66^, who found that the prevalence of metabolic risk factors was higher in rural South African females (25.0%) than in males (10.5%). Moreover, these findings somewhat reflect a gender-specific effect of metabolic risk factors discussed among African-American women precipitating diet-sensitive NCDs^67–69^. In several sub-Saharan African countries^46,70,71^, as well as in the African-American population^17,69^, the higher prevalence of metabolic risk factors in women compared to men has also been identified. However, prevalence estimates from non-African American and other developed nations do not show a clear trend for gender variance in the prevalence of metabolic risk factors^65,69,72^.

Overall, in the context of sub-Saharan Africa, gender differences in health-seeking behaviour, cultural values, healthcare services differences of the study settings, and positive social perceptions surrounding body weight may be attributed to the apparent gender disparities observed in the prevalence of metabolic risk factors^46,73^. For example, an overweight status/ obesity is socially tolerated or even desired in African cultures, and this might be another explanation of the observed gender-variance. Thus, leading to significantly higher levels of obesity and other metabolic risk factors among females compared with males, as observed in the current review and similar to Moradi et al*.*^74^ meta-analysis demonstrating a higher risk of obesity in females when compared to males in food-insecure households (OR 1.26, 95% CI 1.05–1.46). In addition, excess body weight is most recently associated with a lack of the interrelated epidemics of HIV and stigmatisation^65^.

### Strengths and limitations

While there is evidence of an association between food insecurity and metabolic risk factors, it is far from conclusive. The studies included in this review provide insufficient evidence relating to different associations categories; this could be because positive findings are published more often than negative findings. Thus, this review's findings should be interpreted in the context of several strengths and limitations, as per the imprecision and high heterogeneity observed between studies, resulting in low quality of evidence to justify such associations; nevertheless, all findings were judged to be of low risk of bias.

Limitations persist, firstly in the study selection process, with the choice to have a first step in the process that only screens the title is an intermediate step that could miss many articles and the exclusion of qualitative studies. The exclusion of qualitative studies was because the study research question was more epidemiological by design, hence the use of the PEO framework. Qualitative analysis was not eligible to be included in this systematic review; therefore, details may have been excluded. Secondly, since only a small number of included studies was utilised, there was limited opportunity to explore the possible major causes of significant heterogeneity between studies. Moreover, the methods or tools employed across studies to ascertain food insecurity exposure and clinical diagnostic criteria for defining metabolic risk factors varied, and this might be another explanation for the observed heterogeneity among studies.

An important strength of this study is that a study protocol was developed before the conduct of the review and pre-registered with a prospective international register of systematic reviews, and to be made available to the public as a pre-print, thus enhancing our study’s transparency. We adhered exclusively to the *Centre for Reviews and Dissemination* (CRD) guidance for undertaking systematic reviews in healthcare^18^, the *Meta-analysis Of Observational Studies in Epidemiology* (MOOSE)^19^, and the *Preferred Reporting Items for Systematic Reviews and Meta-Analysis* (PRISMA)^20^ guidelines to maximise the rigour and robustness of the methodology used in this study throughout its design, implementation, analysis and reporting.

### Implications for practice

Our central findings that exposure to food insecurity is adversely associated with the presence of metabolic risk factors help define a subgroup at high risk for earlier diet-sensitive NCDs development; thus could have important clinical implications. Firstly, in sub-Saharan Africa, food insecurity is reported to affect approximately more than 257 million (1 in 3) people^4^, one could argue for initiation of regular food insecurity status screening and treatment in clinical settings, as a preventative medicine tool to facilitate early detection and intervention for diet-sensitive NCDs. This strategy is recommended in resource-limited settings, and is endorsed by the Academic Paediatrics Association (APA), the National Academy of Medicine; Centers for Disease Control and Prevention; and the World Health Organization^75,76^ merely because food insecurity status is easily measured without the need of expensive laboratory investigations.

Moreover, our meta-analysis pooled prevalence estimate for metabolic risk factors was found to be 41.8% (95% CI: 33.2% to 50.8%) among the food insecure participants. Such high prevalence of metabolic risk factors among the food insecure participants, warrant clinicians and community health experts to consider follow-up screening for key metabolic risk factors in patients screening positive for food insecurity status, and vice versa, if they are not recent, which could improve the care provided to marginalized populations. However, in the face of limited specialist and resources, the adaptation of a consensus screening checklist may facilitate this goal (paper risk test or anthropometric measurement), primary healthcare workers can be trained to screen for food insecurity status to identify potentially at high-risk individuals for early diagnosis of metabolic risk factors. Thus, also adapting already existing healthcare programmes for other diseases such as HIV/AIDS which are well established in many sub-Saharan African countries for monitoring metabolic risk factors on the causal pathway to diet-sensitive NCDs, may also facilitate this goal.

Secondly, our study findings also suggest an almost twofold female-to-male disparity in the prevalence of key metabolic risk factors among the food insecure, indicating the need for local prioritisation of health resources and interventions to reflect a gender-specific effect.

### Implications for research

Based on the findings of this review, recommendations for future research, many of which are consistent with previous surveys, are presented below:

Our study shows that limited published longitudinal studies have been conducted in sub-Saharan African countries. These research studies are desirable for determining absolute risk, and the causal link between food insecurity exposures, metabolic risk factors, and diet-sensitive NCDs. It is hoped that this study’s results will prompt further primary studies and follow-up studies periodically updating the available evidence and focusing on specific diet-sensitive NCDs such as diabetes to provide contextual insight into the associations across all geographical settings and in both genders.

Lastly, there is a need to confirm the potential prevention benefits of increasing food insecurity status screening and treatment in clinical settings through randomized controlled trials, before it can be recommended for large scale adaptation.

## Conclusion

Our systematic review and meta-analysis provide compelling evidence that exposure to food insecurity is associated with the presence of metabolic risk factors in children (00–14 years), youth (15–24 years), adults (25–64 years), and senior (65 years) participants. Since this is a complex association, high heterogeneity and imprecision culminated in low quality of evidence to support these associations; however, both analyses findings were found to be of low level of bias. In addition, while there is evidence of an association between food insecurity and key metabolic risk factors, the limited number of studies precluded a more in-depth analysis of this association. Thus, these data limitations highlight the need for more rigorous and high-quality longitudinal studies to determine absolute risk, and the causal link between food insecurity exposures, metabolic risk factors, and diet-sensitive NCDs.

Overall, these findings highlight the need to address food insecurity as an integral part of diet-sensitive NCDs prevention programme. Given this approach to food insecurity, health practitioners should be cautioned to screen for food insecurity status and treatment, as preventative medicine tool for metabolic risk factors on the causal pathway to diet-sensitive NCDs. The detection and early treatment of metabolic risk factors as well as associated risk factors, will allow potential diet-sensitive NCDs prevention.

## Supplementary Information

Supplementary Appendix 1.

Supplementary Appendix 2.

Supplementary Appendix 3.

## Data Availability

The data supporting the conclusions of this paper are available through the detailed reference list. No original datasets are presented, due to the fact that this is a review of already existing literature.

## Abbreviations

LMICs: Low- and middle-income countries
MMAT: Mixed methods appraisal tool
PEO: Population, exposure, and outcomes
DSCDs: Diet-sensitive chronic diseases
PRISMA: Preferred reporting items for systematic review and meta-analysis
WHO: World Health Organization
FAO: Food and agriculture organization
NCDs: Non-communicable diseases
