# Supplementary material for: Association between food insecurity and key metabolic risk factors for diet-sensitive non-communicable diseases in sub-Saharan Africa: a systematic review and meta-analysis
Source: Sci Rep. 2021 Mar 4;11:5178. doi: 10.1038/s41598-021-84344-0 (PMC7933340; doi:10.1038/s41598-021-84344-0)
Supplement: Supplementary file 1 — Supplementary Appendix 1. [file 41598_2021_84344_MOESM1_ESM.docx]

**Supplementary Appendix No. 1**

This appendix formed part of the original submission

**Association Between Food Insecurity and Key Metabolic Risk Factors for Diet-Sensitive Non-Communicable Diseases in Sub-Saharan Africa: A Systematic Review and Meta-Analysis**

Sphamandla Josias Nkambule ^1^, Indres Moodley ^1^, Desmond Kuupiel ^1^, Tivani P. Mashamba-Thompson^1,2^

^1^ Department of Public Health Medicine, School of Nursing and Public Health, University of KwaZulu-Natal, Durban, South Africa

^2^ Faculty of Health Sciences, University of Pretoria, Gauteng Province, South Africa

**Corresponding Author:** Mr. Sphamandla Josias Nkambule, B.Ed Hons (Ed Psych), MMedSc

**Address:** Department of Public Health Medicine, School of Nursing and Public Health, University of KwaZulu-Natal, Durban, South Africa

**Email address:** [nkambulesj@gmail.com](mailto:nkambulesj@gmail.com)

**Email addresses of authors:**

Prof. Tivani P. Mashamba-Thompson, MMedSci, PhD [tivani.thompson@gmail.com](mailto:tivani.thompson@gmail.com)

Prof. Indres Moodley, M. Pharm, PhD ([moodleyi15@ukzn.ac.za](mailto:moodleyi15@ukzn.ac.za))

Dr. Desmond Kuupiel, PhD ([desmondkuupiel98@hotmail.com](mailto:desmondkuupiel98@hotmail.com))

**Table of Contents**

**1.** **Changes to the original protocol** 3

**2.** **Search strategies with MeSH descriptors and truncation** 4

**3.** **Results of** **methodological quality (or risk of bias)** **assessment of each included study** 6

**4.** **The overall risk of bias across risk of bias domains** 8

**5.** **The GRADE checklist reasons to up- or down-grade overall quality of evidence** 9

**6.** **The completed GRADE checklist and results interpretation** 10

[**7.** **Full-articles screening, and the Cohen’s Kappa coefficient (κ) statistic results** 12](#_Toc62267473)

[**8.** **Outcome of Interest** 15](#_Toc62267474)

[**a)** **Primary outcomes of interest – Evidence of the associations between food insecurity and key metabolic risk factors** 15](#_Toc62267475)

[**b)** **Secondary outcomes of interest – The prevalence estimates of metabolic risk factors associated with food insecurity among the pooled study participants** 15](#_Toc62267476)

[**9.** **Definition and method of ascertainment for variables (independent and dependent)** 16](#_Toc62267477)

[**10.** **Meta-weighted pooled prevalence results of metabolic risk factors** 19](#_Toc62267478)

[**11.** **Reference List** 20](#_Toc62267479)

1. **Changes to the original protocol**

Please note attached application (See Supplementary Appendix No. 2) for Ethics Approval on the here noted request for amendments on the original protocol proposal:

1. **In our published original protocol, the title of study state:**

The Evidence on the Association Between Food Insecurity and Diet-sensitive Chronic Diseases in Sub-Saharan Africa: A Systematic Review and Meta-analysis

**The amendments requested:**

Association Between Food Insecurity and Key Metabolic Risk Factors for Diet-Sensitive Non-communicable Diseases in Sub-Saharan Africa: A Systematic Review and Meta-Analysis

1. If we observed high heterogeneity between studies, we planned to explore reasons for this in sensitivity analyses. However, few studies were returned, providing little power for detection.
2. We planned to look for publication bias using standard approaches including funnel plots and Egger tests, however, after consulting with an experienced statistician, in the presence of high heterogeneity there is no reason to expect a plot of estimates against their standard errors to have a funnel shape.
3. **Search strategies with MeSH descriptors and truncation**

The PEO framework informed the development of the search strategy since the research question is more epidemiological and also to ensure that the boundaries of the research question are clearly defined:

**Search # 1** – Population (Human participants of all-age groups, residing in sub-Saharan Africa, both genders, and regardless of their ethnic background),

**Search # 2** – Exposure (Food Insecurity), and

**Search # 3** – Outcomes (Diagnosed with any of the following diet-sensitive NCDs metabolic risk factors).

All searches were conducted between 15 May till 28 June 2019.

**Table 1:** Search strategy employed in the review

| **Search** | **Search terms** | **PubMed** | **EBSCOhost^*^** | **Web of Science** | **Ovid^**^ Platform** | **Google Scholar** |
| --- | --- | --- | --- | --- | --- | --- |
| **# 1** | African filter ((((Angola OR Benin OR Botswana OR “Burkina Faso” OR Burundi OR Cameroon OR “Cape Verde” OR “Central African Republic” OR Chad OR Comoros OR Congo OR “Democratic Republic of Congo” OR Djibouti OR “Equatorial Guinea” OR Eritrea OR Ethiopia OR Gabon OR Gambia OR Ghana OR Guinea OR “Guinea Bissau” OR “Ivory Coast” OR “Cote d’Ivoire” OR Kenya OR Lesotho OR Liberia OR Madagascar OR Malawi OR Mali OR Mauritania OR Mauritius OR Mozambique OR Namibia OR Niger OR Nigeria OR Principe OR Reunion OR Rwanda OR “Sao Tome” OR Senegal OR Seychelles OR “Sierra Leone” OR Somalia OR “South Africa” OR Sudan OR Swaziland OR Tanzania OR Togo OR Uganda OR “Western Sahara” OR Zambia OR Zimbabwe OR “Central Africa” OR “Central African” OR “West Africa” OR “West African” OR “Western Africa” OR “Western African” OR “East Africa” OR “East African” OR “Eastern Africa” OR “Eastern African” OR “South African” OR “Southern Africa” OR “Southern African” OR “sub Saharan Africa” OR “sub Saharan African” OR “sub Saharan Africa” OR “sub Saharan African” NOT “guinea pig” NOT “guinea pigs” NOT “aspergillus niger” )))) | 88 799 | 823 050 | 505 656 | N/A | N/A |
| **# 2** | Unhealthy diet OR Food Insecurity OR Food security status OR Food Shortage OR Food insufficiency OR Hunger OR Malnutrition OR nutritional transition OR FI OR Undernutrition OR Food scarcity OR Food OR lack of food OR Fasting OR deprivation of food OR famine OR Poor Nutrition OR Minimum Diet Diversity OR Minimum Adequate Diet OR Minimum Meal Frequency | 152 447 | 345 051 | 99 089 | N/A | N/A |
| **# 3** | Hypertension OR chronic diseases OR Diet-Related OR diet-sensitive chronic diseases OR Diet-Related non-communicable disease OR Diet-Related Chronic Diseases OR non-communicable disease OR Obesity OR Diet-sensitive NCDs OR NCDs OR Underweight OR Overweight OR Dyslipidaemia OR metabolic syndrome OR Metabolic Risk Factors | 137 322 | 72 021 | 32 686 | N/A | N/A |
| **# 4** | # 1 AND # 2 AND # 3  Limits   - Publication Date = 01/01/2015 to 30/08/2019 - Language = No Restrictions - Study Participants = Humans’ studies only | 433 | 1 834 | 20 | 5 756 | 3760 |
| **Total**  **=** **885 exported to EndNote Virtual Library, after title screening** | | 149 | 347 | 14 | 215 | 160 |
| Note: N/A the Platform doesn’t allow multiple search to be ran separately and combine the hits on a new run.  * EBSCOhost (Academic Search Complete, CINAHL with full text, Health Source – Consumer/Nursing/Academic Editions, MedLine with full text, and MedLine),  **Ovid (Journals@Ovid Full Text, and Your Journals@Ovid) | | | | | | |

1. **Results of** **methodological quality (or risk of bias)** **assessment of each included study**

The methodological quality (or risk of bias) of each included study was assessed by two reviewers independently, using the *Mixed Methods Appraisal Tool* (MMAT) – Version 2018,^1^ two core assessment domains and five further assessment domains deemed relevant to appraise the methodological quality of cross-sectional studies.^2^ The summary of the domains critically assessed are provided as supplementary material (Supplementary Appendix No. 2 file). Based on the assessment of each domain, an overall percentage quality score was calculated for each included study (study quality of reporting) and the overall risk of bias across domains. The scores were interpreted as of low quality if ≤ 50 per cent – average quality if 51 – 75 per cent - and high quality if 76 – 100 per cent

The overall quality of evidence (or certainty in the findings) for each outcome collected was assessed based on study methodological quality, results from sensitivity analysis, and by downgrading and upgrading the baseline quality score for cross-sectional studies, according to the domains specified in the *grades of recommendation, assessment, development, and evaluation* (GRADE) guidelines.^3^

**Table 2:** Study quality (or risk of bias) of individual included studies using the Mixed Methods Appraisal Tool (MMAT) – Version 2018 ^1^ adapted for cross-sectional studies

| **First author, Year, Reference** | **Country** | **Screening questions**  **(for all types)** | | **3. Quantitative non-randomised studies** | | | | | **Score* (%)** |
| --- | --- | --- | --- | --- | --- | --- | --- | --- | --- |
|  |  | **S1** | **S2** | **3.1** | **3.2** | **3.3** | **3.4** | **3.5** |  |
| Abebe, 2017 ^4^ | Ethiopia | Yes | Yes | Yes | Yes | Yes | Yes | Yes | 100 |
| Agaba, 2017 ^5^ | Nigeria | Yes | Yes | Yes | Yes | Yes | Yes | Yes | 100 |
| Anteneh, 2015 ^6^ | Ethiopia | Yes | Yes | Yes | Yes | Yes | Yes | Yes | 100 |
| Colecraft, 2018 ^7^ | Ghana | Yes | Yes | Yes | Yes | Yes | No | Yes | 85.7 |
| Cox, 2016 ^8^ | Malawi | Yes | Yes | Yes | No | Can’t tell | Yes | Yes | 100 |
| Desalew, 2017 ^9^ | Ethiopia | Yes | Yes | Yes | Yes | Yes | No | Yes | 85.7 |
| Di Gioia, 2016 ^10^ | Madagascar | Yes | Yes | Yes | Yes | Yes | Can’t tell | Yes | 71 |
| Gebremichael, 2019 ^11^ | Ethiopia | Yes | Yes | Yes | Yes | Yes | Yes | Yes | 100 |
| Soubeiga, 2017 ^12^ | Burkina Faso | Yes | Yes | Yes | Yes | Yes | Yes | Yes | 100 |
| Katalambula, 2018 ^13^ | Tanzania | Yes | Yes | Yes | Yes | Yes | Yes | Yes | 100 |
| Kejo, 2018 ^14^ | Tanzania | Yes | Yes | Yes | Yes | Yes | Can’t tell | Yes | 85.7 |
| Lapauw, 2016 ^15^ | Ghana | Yes | Yes | Yes | Can’t tell | Can’t tell | Can’t tell | Yes | 57.1 |
| Maimela, 2016 ^16^ | South Africa | Yes | Yes | Yes | Yes | Yes | No | Yes | 85.7 |
| Mbaissouroum, 2017 ^17^ | South Africa | Yes | Yes | Yes | Yes | Can’t tell | No | Yes | 71.4 |
| Mohammed, 2016 ^18^ | Ghana | Yes | Yes | Yes | Yes | Yes | No | Yes | 85.7 |
| Musaiger, 2016 ^19^ | Sudan | Yes | Yes | Can’t tell | Yes | Yes | Can’t tell | Yes | 71.4 |
| Musaiger, 2016 ^20^ | Sudan | Yes | Yes | Yes | Yes | Yes | No | Yes | 85.7 |
| Mutisya, 2015 ^21^ | Kenya | Yes | Yes | Yes | Yes | Yes | No | Yes | 71 |
| Solomons, 2018 ^22^ | South Africa | Yes | Yes | Yes | Can’t tell | Yes | No | Yes | 71.4 |
| Omech, 2016 ^23^ | Botswana | Yes | Yes | Yes | Yes | Can’t tell | Can’t tell | Yes | 71.4 |
| Nansseu, 2019 ^24^ | Cameroon | Yes | Yes | Yes | Yes | Yes | Yes | Yes | 100 |
| Tateyama, 2018 ^25^ | Zambia | Yes | Yes | Yes | Yes | Yes | Yes | Yes | 100 |
| **The overall risk of bias across domains** | | 22 | 22 | 21 | 19 | 18 | 9 | 22 | 86.4 |

* meets % of the MMAT criteri

1. **The overall risk of bias across risk of bias domains**

***Table 3*** provides the review authors’ judgements of additional consideration on potential sources of bias. Overall, the majority of included studies in the present systematic review were found to have low to unclear risk of bias from the *Mixed Methods Appraisal Tool* (MMAT),^1^ critically assessed risk of bias domains.

In the confounding assessment (risk of bias domain 6), 8 studies were found to have high risk of bias and in the measurements appropriate regarding both the outcome and intervention (or exposure) assessment (risk of bias domain 4), 1 study was found to have high risk of bias. Sensitivity meta-analysis was performed after excluding these studies.

**Table 3:** Risk of bias: review authors’ judgements about each risk of bias domain across all included studies.

| **The *Mixed Methods Appraisal Tool* (MMAT),^1^ risk of bias domains** | **Low risk of bias**  **n/N (%)** | **Unclear risk of bias**  **n/N (%)** | **High risk of bias**  **n/N (%)** |
| --- | --- | --- | --- |
| 1. **Are there clear research questions?** | 22/22 (100) | 0/22 (0) | 0/22 (0) |
| 1. **Do the collected data allow to address the research questions?** | 22/22 (100) | 0/22 (0) | 0/22 (0) |
| 1. **Are the participants representative of the target population?** | 21/22 (95.5) | 1/22 (4.5) | 0/22 (0) |
| 1. **Are measurements appropriate regarding both the outcome and intervention (or exposure)?** | 19/22 (86.4) | 2/22 (9.0) | 1/22 (4.5) |
| 1. **Are there complete outcome data?** | 18/22 (81.8) | 4/22 (18.1) | 0/22 (0) |
| 1. **Are the confounders accounted for in the design and analysis?** | 9/22 (40.9) | 5/22 (22.7) | 8/22 (36.4) |
| 1. **During the study period, is the intervention administered (or exposure occurred) as intended?** | 22/22 (100) | 0/22 (0) | 0/22 (0) |

1. **The GRADE checklist reasons to up- or down-grade overall quality of evidence**

In the *grades of recommendation, assessment, development, and evaluation* (GRADE) guidelines,^3^ non-randomised studies provide low quality evidence for outcomes at the beginning of the quality assessment. Our confidence in the evidence for an outcome can then be up- or down-graded according to different aspects of evidence quality. See the supplementary information for further detail on up- or down-grading below:

**Reasons to up- or down-grade quality of evidence in GRADE**

**Downgrade Reasons Include:**

1. **Risk of bias:**

Not serious if >50% of studies have no domain which is at high risk of bias.

- Serious if studies are judged to be between ‘not serious’ and ‘very serious’.

- Very serious if studies which have two or more domains at high risk of bias represent more than 50% of the total studies and contribute more than 50% to any meta-analyses.

1. **Inconsistency:**

Not serious if have 0, serious if have 1, and very serious if have two or more of the following:

**-** Heterogeneity is moderate (I² ~30-60%), or above.

**-** Wide variance of point estimates across different studies.

**-** Minimal overlap of confidence intervals.

1. **Indirectness:**

Not serious if have 0, serious if have 1, and very serious if have two of the following:

- An indirect comparison (for example study A compares to a placebo and study B compares to a different drug).

- Studies differ in terms of population (e.g. hospital inpatients only vs population-based survey)

- Studies differ in terms of exposure definition (e.g. for example use different methods to food insecurity exposures).

- Studies differ in terms of outcome measures (e.g. for example be restricted to certain time-frames or have a different definition of a Metabolic risk outcome).

1. **Imprecision:**

Not serious, or serious if, and very serious if the following:

- Serious imprecision: Wide confidence intervals

- Very serious imprecision: Very wide confidence intervals

**Upgrading Reasons Include:**

1. **Large effect:**

- None: most effect estimates <2

- Strong association: effect estimates >2 or <0.5 (based on direct evidence, with no plausible confounders)

- Very strong association: effect estimates >4 or <0.2 (based on direct evidence with no serious problems with risk of bias or precision, i.e. with (sufficiently narrow confidence intervals).

1. **Plausible confounding:**

- Would dilute the demonstrated effect: e.g. If, for instance, only sicker patients receive an intervention or exposure, yet they still fare better, it is likely that the actual intervention or exposure effect is even larger than the data suggest (confounding by indication).

- Would suggest spurious effect: When confounding is expected to increase the effect, but no effect was observed.

1. **Dose response gradient:**

- Yes

- No

1. **The completed GRADE checklist and results interpretation**

***Table 4*** provides our GRADE assessment results, the review authors’ judgements for each metabolic risk factor outcomes. Obesity, Dyslipidaemia, Hypertension, Underweight, and Overweight demonstrated inconsistency (or high heterogeneity). It is important to note that heterogeneity is poorly estimated where there are few studies. Obesity, Dyslipidaemia, Hypertension, Underweight, and Overweight demonstrated Very Serious Indirectness (because of indirectness of population, outcome and exposure). The methods or tools employed across studies to assess food insecurity exposure and clinical diagnostic criteria for defining metabolic risk factors varied, and this might be another explanation for the observed heterogeneity among studies

**Table 4:** The completed GRADE checklist overall quality assessment

| **QUALITY ASSESSMENT** | | | | | | | |
| --- | --- | --- | --- | --- | --- | --- | --- |
| **Outcome** | **Downgrade** | | | | |  | **Quality** |
|  | **Number of studies and Study Design** | **Risk of bias** | **Inconsistency** | **Indirectness** | **Imprecision** | **Publication bias** |  |
| Obesity | 12 Cross-sectional | Not Serious | Very Serious | Very Serious Indirectness (because of indirectness of population, outcome and exposure) | Serious Imprecision | Very Serious (because asymmetrical shape of the graph) | **⊕**⊕⊕⊕  Very low |
| Dyslipidaemia | 3 Cross-sectional | Not Serious | Serious | Very Serious Indirectness (because of indirectness of population, outcome and exposure) | Very Serious Imprecision | * | **⊕**⊕⊕⊕  Very low |
| Hypertension | 12 Cross-sectional | Not Serious | Serious | Very Serious Indirectness (because of indirectness of population, outcome and exposure) | Very Serious Imprecision | Very Serious (because asymmetrical shape of the graph) | **⊕**⊕⊕⊕  Very low |
| Underweight | 6 Cross-sectional | Not Serious | Very Serious | Very Serious Indirectness (because of indirectness of population, outcome and exposure) | Serious Imprecision | * | **⊕**⊕⊕⊕  Very low |
| Overweight | 7 Cross-sectional | Not Serious | Very Serous | Very Serious Indirectness (because of indirectness of population, outcome and exposure) | Serious Imprecision | * | **⊕⊕**⊕⊕  Very ow |
| Acute Respiratory Infections (ARI) | 1 Cross-sectional | Not Serious | * | * | * | * | **⊕⊕**⊕⊕  Low |
| left ventricular mass | 1 Cross-sectional | Not Serious | * | * | * | * | **⊕⊕**⊕⊕  Low |
| Stunting | 2 Cross-sectional | Not Serious | * | Not Serious | * | * | **⊕⊕**⊕⊕  Low |

**Explanatory Notes:**

* Due to small number of studies (n<10) could not be formally assessed.

1. **Full-articles screening, and the Cohen’s Kappa coefficient (κ) statistic results**

After the full-article screening stage, we measured the degree of the agreement between reviewers using the Cohen’s Kappa coefficient (κ) statistic, Stata 13.0SE (StataCorp College Station, TX, USA) a robust statistic used for inter-rater reliability testing.^26^ Interpretation of the results below:

We calculated degree of agreement following full article screening. The results showed that there was 92.47% agreement versus 62.23% expected by chance which constitutes a considerably high agreement between screeners (Kappa statistic = - 0. 80 and p-value <0.05). In addition, the McNemar’s chi-square statistic suggests that there is no statistically significant difference in the proportions of yes/no answers by reviewers with p-value >0.05.

**Table 5:** Full-article screening results

| **Author and Date** | **Screener One (PI)** | **Co-Screener (1^st^ Author)** |
| --- | --- | --- |
| Abebe, 2017 | 1 | 1 |
| Abubakari, 2015 | 1 | 0 |
| Abusalma, 2015 | 1 | 1 |
| Ackatia-Armah, 2015 | 1 | 0 |
| Adejumo, 2016 | 1 | 0 |
| Agaba, 2017 | 1 | 1 |
| Ahmed, 2018 | 1 | 1 |
| Alicke, 2017 | 1 | 1 |
| Altare, 2016 | 1 | 1 |
| Amare, 2015 | 1 | 1 |
| Anteneh, 2015 | 1 | 1 |
| Audain, 2017 | 0 | 1 |
| Aworh, 2018 | 0 | 1 |
| Barich, 2018 | 1 | 1 |
| Benzekri, 2015 | 0 | 1 |
| Boateng, 2019 | 1 | 1 |
| Cabral, 2019 | 0 | 0 |
| Carruth, 2019 | 1 | 1 |
| Cockx, 2018 | 0 | 0 |
| Cox, 2017 | 1 | 1 |
| Craveiro, 2016 | 1 | 1 |
| Daivadanam, 2019 | 0 | 1 |
| Danquah, 2018 | 1 | 1 |
| De Vita, 2019 | 1 | 1 |
| Demaio, 2018 | 0 | 0 |
| Desalew, 2017 | 1 | 1 |
| Di Gioia, 2016 | 1 | 1 |
| Doumit, 2015 | 0 | 0 |
| Ebbeling, 2018 | 0 | 0 |
| El Kabbaoui, 2018 | 1 | 1 |
| Faber, 2016 | 0 | 0 |
| Fekadu, 2015 | 1 | 1 |
| Gebremariam, 2018 | 0 | 0 |
| Gebremichael, 2019 | 1 | 1 |
| Gebreselassie, 2015 | 1 | 1 |
| Gebrihet, 2017 | 1 | 1 |
| Grellety, 2017 | 1 | 1 |
| Guwatudde, 2015 | 1 | 1 |
| Gyamea, 2018 | 1 | 1 |
| Holmes, 2018 | 1 | 1 |
| Humphries, 2015 | 1 | 1 |
| Hunter-Adams, 2019 | 0 | 0 |
| Jannasch, 2017 | 1 | 1 |
| Jones, 2016 | 1 | 1 |
| Kakota, 2015 | 0 | 0 |
| Katalambula, 2018 | 1 | 1 |
| Kejo, 2018 | 1 | 1 |
| Koma, 2017 | 1 | 1 |
| Korkalo, 2015 | 0 | 0 |
| Krasevec, 2017 | 1 | 1 |
| Lapauw, 2016 | 1 | 1 |
| Lelijveld, 2016 | 1 | 1 |
| M’Kaibi, 2017 | 1 | 1 |
| Mahgoub, 2017 | 0 | 0 |
| Maimela, 2016 | 1 | 1 |
| Maiyoh, 2019 | 1 | 1 |
| Maketa, 2015 | 0 | 0 |
| Mbaissouroum, 2017 | 1 | 1 |
| Melaku, 2016 | 1 | 1 |
| Melaku, 2018 | 1 | 1 |
| Melaku, 2018 | 1 | 1 |
| Mohammed, 2016 | 1 | 1 |
| Molyneux, 2018 | 0 | 0 |
| Moore, 2016 | 1 | 1 |
| Motbai0r, 2015 | 1 | 1 |
| Musaiger, 2016 | 1 | 1 |
| Musaiger, 2016 | 1 | 1 |
| Mutisya, 2015 | 1 | 1 |
| Mwenda, 2018 | 0 | 0 |
| Neupane, 2015 | 1 | 1 |
| Obirikorang, 2015 | 1 | 1 |
| Olaitan, 2018 | 1 | 1 |
| Padrão, 2015 | 1 | 1 |
| Peer, 2015 | 1 | 1 |
| Peer, 2016 | 1 | 1 |
| Raji, 2015 | 1 | 1 |
| Solomons, 2018 | 1 | 1 |
| Steenkamp, 2016 | 1 | 1 |
| Stobaugh, 2018 | 1 | 1 |
| Tadesse, 2017 | 1 | 1 |
| Trehan, 2015 | 0 | 0 |
| Van Der Kam, 2016 | 0 | 0 |
| Van Der Linden, 2019 | 1 | 1 |
| Wekesah, 2018 | 1 | 1 |
| Wesonga, 2016 | 1 | 1 |
| Whyte, 2016 | 0 | 0 |
| Willie, 2018 | 0 | 0 |
| Wilson, 2018 | 0 | 0 |
| Wu, 2015 | 1 | 1 |
| Yaya, 2018 | 1 | 1 |
| Yaya, 2018 | 1 | 1 |
| Ze, 2018 | 1 | 1 |
| Zlotnick, 2015 | 0 | 0 |

**Note:** Yes = 1

No = 0

**Stata output**

Expected

Agreement Agreement Kappa Std. Err. Z Prob>Z

-----------------------------------------------------------------

92.47% 62.23% 0.8007 0.1037 7.73 0.0000

. mcc ScreenerOnePI CoScreener

| Controls |

Cases | Exposed Unexposed | Total

-----------------+------------------------+------------

Exposed | 66 3 | 69

Unexposed | 4 20 | 24

-----------------+------------------------+------------

Total | 70 23 | 93

McNemar's chi2(1) = 0.14 Prob > chi2 = 0.7055

Exact McNemar significance probability = 1.0000

Proportion with factor

Cases .7419355

Controls .7526882 [95% Conf. Interval]

--------- --------------------

difference -.0107527 -.0772214 .0557161

ratio .9857143 .9148426 1.062076

rel. diff. -.0434783 -.2737873 .1868308

odds ratio .75 .1098635 4.43326 (exact)

1. **Outcome of Interest**
2. **Primary outcomes of interest – Evidence of the associations between food insecurity and key metabolic risk factors**

For the current systematic review, we aimed to answer the question, “What are the associations between food insecurity and key metabolic risk factors for diet-sensitive NCDs in sub-Saharan African population?” Considering the question, the primary outcomes of interest were key metabolic risk factors, including Obesity, Dyslipidaemia, Hypertension, Underweight, and Overweight independent of socio-demographic and lifestyle factors.

Following extraction of the relevant data, we noted the need to stratify the studies in three associations strata, namely:

1. **[-] Adverse Association** – Food insecurity was reported to be significantly associated with ≥ one key metabolic risk factor (observed with relevant statistical tool);
2. **[+] Non-significant Association** – There was no significant association found between food insecurity and reported key metabolic risk factors (observed with relevant statistical tool), and
3. **[~] Inconclusive Association** – The association between food insecurity and reported metabolic risk outcomes were inconclusive because, although exposure to food insecurity showed a protective effect toward reported metabolic risk factors, it was not statistically significant (observed with relevant statistical tool).
4. **Secondary outcomes of interest – The prevalence estimates of metabolic risk factors associated with food insecurity among the pooled study participants**

The secondary outcome measure, in this meta-analysis, is the prevalence of metabolic risk factors associated with food insecurity in sub-Saharan Africa. It is aimed to assess the pooled estimates of metabolic risk factors on the causal pathway to diet-sensitive NCDs patterned by food insecurity among the food insecure population in sub-Saharan Africa and lastly to visualise the variation patterns in the occurrence of these metabolic risk factors by gender and geographic area.

1. **Definition and method of ascertainment for variables (independent and dependent)**

Studies reporting any one of the food insecurity methods of ascertainment/measure of food insecurity exposure listed in Table 1 as an independent variable, were included in this review.

**Table 6:** Definition of recoded food insecurity method of ascertainment

| **Criteria** | **Exposure definition** | **The theoretical minimum risk exposure level** |
| --- | --- | --- |
| Minimum Diet Diversity ^[[1]](#footnote-1)^ | The consumption of four or more food groups from the seven food groups. | The proportion of eating from less than four food groups, due to inconsistent access to food |
| Minimum Adequate Diet ^1^ | Average daily consumption of more than five servings of fruits and vegetables which is equivalent to at least 290–430 g per day | Consumption of fewer than five servings of fruits and vegetables, due to not enough money for food |
| Minimum Meal Frequency ^1^ | The proportion of the population who eat the recommended minimum number of meals in a day. Stratified by age | Two times for breastfed infants aged 6 to 8 months; three times for breastfed children aged 9 to 23 months; four times for non-breastfed children aged ≥ 23 months |
| Food Insecurity Access Scale ^2^ | The Household Food Insecurity Access Scale (HFIAS) Indicator Guide V.3 and categorised into four levels | Food secure, mildly food insecure, moderately food insecure, or severely food insecure. |

Food insecurity is inherently unobservable and challenging to define, but both intrinsically and instrumentally crucial to public health. Across the included studies in this review, food insecurity exposure was generally measured either at a household level or at an individual level:

1. At the household level, valid food insecurity measures (instruments), assess household access to food, which include the Household Food Insecurity Access Scale (six-item short questionnaire), expenditure and consumption studies (dietary intake), and dietary diversity measures; yet individuals experience food insecurity.
2. Thus, at an individual level, valid food insecurity measures (instruments) assess utilisation which captures individual food insecurity through the use of food intake measures, dietary diversity scores, and assessments of caring practices (feeding behaviour) are common alternative increasingly employed by food security and nutrition analysts. Another strategy is to employ anthropometric indicators/ or measures such as weight-for-height, or height-for-age Z-scores – the former indicating “wasting” or short-term nutritional deprivation, the latter indicating “stunting” or long-term undernutrition – as well as weight-for-age, body mass index and mid-upper arm circumference. The first three measures are only intended to assess children’s nutritional status, typically those under 60 months of age.

**Metabolic Risk Factors** - This refers to any attribute, characteristic, or exposure of an individual, which increases the likelihood of developing a disease or other unwanted condition/event. Metabolic risk factors double a person’s risk of diet-sensitive NCDs, such as blood vessels and heart disease, leading to heart attacks and strokes. They increase a person’s risk of diabetes by five times.

Studies reporting at least one of the major metabolic risk factors (see Table 2) as a dependent variable, diagnosed according to either one of the international diagnostic criteria were considered for inclusion: NCEP-ATPIII (2001),^27^ International Diabetes Foundation (IDF 2005),^28^ AHA/NHLBI criteria (2004),^29^ and any other measures in line with the World Health Organization criteria (1998)^30^ extensively used to diagnose metabolic risk factors.

**Table 7:** Several definitions of diagnostic criteria of the review outcomes

| **Risk factors** | **Definition** | **The criteria for diagnosis^[[2]](#footnote-2)^** |
| --- | --- | --- |
| Obesity | The abnormal or excessive fat accumulation  Large Waist Size | Body mass index 30 kg/m2 and/or  waist: hip ratio (men > 0.9, women > 0.85), For men: 40 inches or larger; For women: 35 inches or larger |
| Dyslipidaemia | Elevation of plasma cholesterol, triglycerides (TGs), or both, or a low HDL cholesterol level that contributes to the development of atherosclerosis | Lipoprotein-Cholesterol – HDL-cholesterol (male < 1.03 mmol/L (40 mg/dL), women < 1.29 mmol/L (50 mg/dL))  Hypertriglyceridemia – Raised triglyceride (≥ 1.7 mmol/L (150 mg/dL) |
| Hypertension | Defined as ≥140/90/90 mm Hg, systolic and diastolic pressure | Optimal blood pressure – <120/80 mm Hg, systolic and diastolic blood pressure  Prehypertension – 120–139/80–89 mm Hg, systolic and diastolic blood pressure  Stage 1 hypertension – 140–159/90–99 mm Hg, systolic and diastolic blood pressure  Stage 2 hypertension – > 160/100 mm Hg, systolic and diastolic blood pressure |
| Underweight and Overweight | Overweight can be defined as excessive and abnormal fat depositions in our bodies. | If body mass index = Underweight (<18.5); normal ([18.5–25[); overweight ([25–30[); obese (≥30) |

1. **Meta-weighted pooled prevalence results of metabolic risk factors**

**Table 7:** Meta-weighted pooled prevalence results of metabolic risk factors with included studies

| Region | Country | Author, Year | Total Participants | | MetS Cases | | Prevalence (95% CI) |
| --- | --- | --- | --- | --- | --- | --- | --- |
|  |  |  | Male | Female | Male | Female |  |
| Central Africa | Cameroon | Nansseu, 2019 ^24^ | 501 | 430 | 134 | 173 | 0.33 (0.30, 0.36) |
| Western Africa | Ghana | Mohammed, 2016 ^18^ | 90 | 90 | 52 | 72 | 0.69 (0.62, 0.75) |
|  | Ghana | Lapauw, 2016 ^15^ | 0 | 250 | 0 | 186 | 0.65 (0.59, 0.71) |
|  | Burkina Faso | Soubeiga, 2017 ^12^ | 2230 | 2399 | 1114 | 1181 | 0.50 (0.48, 0.51) |
|  | Ghana | Colecraft, 2018 ^7^ | 513 | 652 | 28 | 35 | 0.05 (0.04, 0.07) |
|  | Nigeria | Agaba, 2017 ^5^ | 521 | 362 | 254 | 199 | 0.51(0.48, 0.55) |
| Southern Africa | Botswana | Omech, 2016 ^23^ | 75 | 216 | 58 | 227 | 0.56 (0.50, 0.62) |
|  | South Africa | Solomons, 2018 ^22^ | 113 | 341 | 60 | 172 | 0.51 (0.46, 0.56) |
|  | South Africa | Mbaissouroum, 2017 ^17^ | 879 | 1266 | 225 | 569 | 0.37 (0.35, 0.39) |
|  | South Africa | Maimela, 2016 ^16^ | 525 | 878 | 337 | 865 | 0.84 (0.82, 0.85) |
| Eastern Africa | Zambia | Tateyama, 2018 ^25^ | 335 | 354 | 169 | 183 | 0.51 (0.47, 0.55) |
|  | Kenya | Mutisya, 2015 ^21^ | 3462 | 3396 | 1858 | 1491 | 0.49 (0.48, 0.50) |
|  | Sudan | Musaiger, 2016 ^19^ | 507 | 438 | 46 | 54 | 0.11 (0.09, 0.13) |
|  | Sudan | Musaiger, 2016 ^20^ | 183 | 217 | 58 | 88 | 0.37 (0.32, 0.41) |
|  | Tanzania | Kejo, 2018 ^14^ | 242 | 194 | 155 | 258 | 0.78 (0.74, 0.82) |
|  | Tanzania | Katalambula, 2018 ^13^ | 684 | 766 | 239 | 276 | 0.36 (0.33, 0.38) |
|  | Ethiopia | Gebremichael, 2019 ^11^ | 156 | 164 | 92 | 76 | 0.53 (0.47, 0.58) |
|  | Madagascar | Di Gioia, 2016 ^10^ | 147 | 166 | 63 | 61 | 0.40 (0.34, 0.45) |
|  | Ethiopia | Desalew, 2017 ^9^ | 187 | 261 | 61 | 85 | 0.33 (0.28, 0.37) |
|  | Malawi | Cox, 2016 ^8^ | 517 | 311 | 45 | 24 | 0.08 (0.07, 0.10) |
|  | Ethiopia | Anteneh, 2015 ^6^ | 177 | 254 | 22 | 50 | 0.17 (0.13, 0.20) |
|  | Ethiopia | Abebe, 2017 ^4^ | 409 | 751 | 137 | 261 | 0.34 (0.32, 0.37) |

1. **Reference List**

1. Hong QN, Pluye P, Fàbregues S, Bartlett G, Boardman F, Cargo M, et al. Mixed methods appraisal tool (MMAT), version 2018. IC Canadian Intellectual Property Office, Industry Canada. 2018.

2. Higgins JP, Sterne JA, Savovic J, Page MJ, Hróbjartsson A, Boutron I, et al. A revised tool for assessing risk of bias in randomised trials. Cochrane database of systematic reviews. 2016;10(Suppl 1):29-31.

3. Guyatt GH, Oxman AD, Vist GE, Kunz R, Falck-Ytter Y, Alonso-Coello P, et al. GRADE: an emerging consensus on rating quality of evidence and strength of recommendations. Bmj. 2008;336(7650):924-6.

4. Abebe SM, Andargie G, Shimeka A, Alemu K, Kebede Y, Wubeshet M, et al. The prevalence of non-communicable diseases in northwest Ethiopia: survey of Dabat Health and Demographic Surveillance System. BMJ Open. 2017;7(10):e015496.

5. Agaba E, I, Maxwell OA, Edith NO, Patricia AA, Amaka NO, Zumnan MG, et al. A survey of non- communicable diseases and their risk factors among university employees: a single institutional study. Cardiovascular journal of Africa. 2017;28(6):377.

6. Anteneh ZA, Gedefaw M, Tekletsadek KN, Tsegaye M, Alemu D. Risk Factors of Overweight and Obesity among High School Students in Bahir Dar City, North West Ethiopia: School Based Cross- Sectional Study. Advances in Preventive Medicine. 2015;2015:1-9.

7. Colecraft EK, Asante M, Christian AK, Adu-Afarwuah S. Sociodemographic Characteristics, Dietary Practices, and Nutritional Status of Adults with Hypertension in a Semi-Rural Community in the Eastern Region of Ghana. International Journal of Hypertension. 2018;2018:1-7.

8. Cox M, Rose L, Kalua K, De Wildt G, Bailey R, Hart J. The prevalence and risk factors for acute respiratory infections in children aged 0-59 months in rural Malawi: A cross-sectional study. Influenza and Other Respiratory Viruses. 2017;11(6):489-96.

9. Desalew A, Mandesh A, Semahegn A. Childhood overweight, obesity and associated factors among primary school children in dire dawa, eastern Ethiopia; a cross-sectional study. BMC Obesity. 2017;4(1).

10. Di Gioia G, Creta A, Fittipaldi M, Giorgino R, Quintarelli F, Satriano U, et al. Effects of Malnutrition on Left Ventricular Mass in a North-Malagasy Children Population. PLOS ONE. 2016;11(5):e0154523.

11. Gebremichael GB, Berhe KK, Zemichael TM. Uncontrolled hypertension and associated factors among adult hypertensive patients in Ayder comprehensive specialised hospital, Tigray, Ethiopia, 2018. BMC Cardiovascular Disorders. 2019;19(1).

12. Soubeiga JK, Millogo T, Bicaba BW, Doulougou B, Kouanda S. Prevalence and factors associated with hypertension in Burkina Faso: a countrywide cross-sectional study. BMC Public Health. 2017;17(1).

13. Katalambula L, Petrucka P, Buza J, Ngoma T. Colorectal Cancer Epidemiology in Tanzania: Patterns in Relation to Dietary and Lifestyle Factors. American Society of Clinical Oncology; 2018.

14. Kejo D, Mosha TCE, Petrucka P, Martin H, Kimanya ME. Prevalence and predictors of undernutrition among underfive children in Arusha District, Tanzania. Food Science & Nutrition. 2018;6(8):2264-72.

15. Lapauw B. DIETARY PATTERN AND METABOLIC SYNDROME AMONG URBAN SLUM WOMEN, ACCRA GHANA: University of Health and Allied Sciences; 2016.

16. Maimela E, Alberts M, Modjadji SEP, Choma SSR, Dikotope SA, Ntuli TS, et al. The Prevalence and Determinants of Chronic Non-Communicable Disease Risk Factors amongst Adults in the Dikgale Health Demographic and Surveillance System (HDSS) Site, Limpopo Province of South Africa. PLOS ONE. 2016;11(2):e0147926.

17. Mbaissouroum M. Risk Factors of High Blood Pressure in Older South Africans: Southern Connecticut State University; 2017.

18. Mohammed H, Ghosh S, Vuvor F, Mensah-Armah S, Steiner-Asiedu M. Dietary intake and the dynamics of stress, hypertension and obesity in a periurban community in Accra. Ghana Medical Journal. 2016;50(1):16.

19. Musaiger AO, Nabag FO, Al-Mannai M. Obesity, Dietary Habits, and Sedentary Behaviors Among Adolescents in Sudan. Food and Nutrition Bulletin. 2016;37(1):65-72.

20. Musaiger AO, Al-Khalifa F, Al-Mannai M. Obesity, unhealthy dietary habits and sedentary behaviors among university students in Sudan: growing risks for chronic diseases in a poor country. Environmental health and preventive medicine. 2016;21(4):224-30.

21. Mutisya M, Kandala N-B, Ngware MW, Kabiru CW. Household food (in)security and nutritional status of urban poor children aged 6 to 23 months in Kenya. BMC Public Health. 2015;15(1).

22. Solomons N, Kruger HS, Puoane T. Association between dietary adherence, anthropometric measurements and blood pressure in an urban black population, South Africa. South African Journal of Clinical Nutrition. 2018:1-9.

23. Omech B, Tshikuka J-G, Mwita J, Tsima B, Nkomazana O, Amone_P’ Olak K. Prevalence and determinants of metabolic syndrome: a cross-sectional survey of general medical outpatient clinics using National Cholesterol Expanded Program-Adult Treatment Panel III criteria in Botswana. Diabetes, Metabolic Syndrome and Obesity: Targets and Therapy. 2016;Volume 9:273-9.

24. Nansseu JR, Kameni BS, Assah FK, Bigna JJ, Petnga S-J, Tounouga DN, et al. Prevalence of major cardiovascular disease risk factors among a group of sub-Saharan African young adults: a population-based cross-sectional study in Yaoundé, Cameroon. BMJ Open. 2019;9(10):e029858.

25. Tateyama Y, Techasrivichien T, Musumari PM, Suguimoto SP, Zulu R, Macwan’Gi M, et al. Obesity matters but is not perceived: A cross-sectional study on cardiovascular disease risk factors among a population-based probability sample in rural Zambia. PLOS ONE. 2018;13(11):e0208176.

26. McHugh ML. Interrater reliability: the kappa statistic. Biochemia medica: Biochemia medica. 2012;22(3):276-82.

27. Detection NCEPEPo, Adults ToHBCi. Third report of the National Cholesterol Education Program (NCEP) Expert Panel on detection, evaluation, and treatment of high blood cholesterol in adults (Adult Treatment Panel III): International Medical Pub; 2002.

28. Zimmet P, Alberti KGM, Serrano Ríos M. A new international diabetes federation worldwide definition of the metabolic syndrome: the rationale and the results. Revista Española de Cardiología (English Edition). 2005;58(12):1371-5.

29. Grundy S, Brewer Jr H, Cleeman J, Smith Jr S, Lenfant C. National Heart L, et al. Definition of metabolic syndrome: report of the National Heart, Lung, and Blood Institute/American Heart Association conference on scientific issues related to definition. Arterioscler Thromb Vasc Biol. 2004;24(2):e13-8.

30. Alberti KGMM, Zimmet PZ. Definition, diagnosis and classification of diabetes mellitus and its complications. Part 1: diagnosis and classification of diabetes mellitus. Provisional report of a WHO consultation. Diabetic medicine. 1998;15(7):539-53.

1. The key measures of Food Insecurity for this review are adopted from FAO, I., UNICEF, WFP, WHO. (2019). The state of food security and nutrition in the world 2019. Safeguarding against economic slowdowns and downturns: FAO Rome (Italy). [↑](#footnote-ref-1)
2. Criteria for diagnosis is adopted from the World Health Organization. (2010) STEPwise Approach to Chronic Disease Risk Factor Surveillance (STEPS). <http://www.who.int/chp/steps/riskfactor/en/index.html> (accessed Oct 2019). [↑](#footnote-ref-2)
