# Supplementary material for: Association between food insecurity and key metabolic risk factors for diet-sensitive non-communicable diseases in sub-Saharan Africa: a systematic review and meta-analysis
Source: Sci Rep. 2021 Mar 4;11:5178. doi: 10.1038/s41598-021-84344-0 (PMC7933340; doi:10.1038/s41598-021-84344-0)
Supplement: Supplementary file 2 — Supplementary Appendix 2. [file 41598_2021_84344_MOESM2_ESM.pdf]

Supplementary Appendix No. 2

This appendix formed part of the original submission

Associations Between Food Insecurity, and Key Metabolic Risk Factors for Diet-Sensitive Non-communicable Diseases in Sub-Saharan Africa: A Systematic Review and Meta-Analysis

Sphamandla Josias Nkambule [1], Indres Moodley [1], Desmond Kuupiel [1], Tivani P. Mashamba-Thompson[1,2]

[1] Department of Public Health Medicine, School of Nursing and Public Health, University of KwaZulu-Natal, Durban, South Africa

[2]Faculty of Health Sciences, University of Pretoria, Gauteng Province, South Africa

Corresponding Author: Mr. Sphamandla Josias Nkambule, B.Ed Hons (Ed Psych), MMedSc

Address: Department of Public Health Medicine, School of Nursing and Public Health, University of KwaZulu-Natal, Durban, South Africa

Email address: nkambulesj@gmail.com

Email addresses of authors:

Prof. Tivani P. Mashamba-Thompson, MMedSci, PhD tivani.thompson@gmail.com

Prof. Indres Moodley, M. Pharm, PhD (moodleyi15@ukzn.ac.za)

Dr. Desmond Kuupiel, PhD (desmondkuupiel98@hotmail.com)

Table of Contents

1. **Three sets of screening checklist/tools – Google Forms**.. 3
2. **The Mixed Methods Appraisal Tool (MMAT) – Version 2018**... 19
3. **Ethical Approval Letter**... 20
4. **Request for Ethical Approval Amendment – Title of Study**.. 21

The evidence on the association between FI and Key Metabolic Risk Factors for DSCDs in Sub-Saharan Africa: A Systematic Review

Title & Abstract Level 1 Screening Form

1. Author and Date

____

2. Title

____

____

____

____

____

3. Was this research article published between January 2015 - October 2019?

Mark only one oval.

- ⬭ Yes
- ⬭ No

4. Does this research article present an abstract?

Mark only one oval.

- Yes
- No

5. Does this research study present evidence from sub-Saharan Africa?

Mark only one oval.

- Yes
- No

6. Does this research study present evidence on Food Insecurity?

Mark only one oval.

- Yes
- No

7. Does this research study present evidence on Obesity?

Mark only one oval.

- Yes
- No

8. Does this research study present evidence on Diabetes?

Mark only one oval.

- Yes
- No

9. Does this research study present evidence on Cardiovascular diseases?

Mark only one oval.

- Yes
- No

10. Does this research study present evidence on stunting, and underweight or wasting?

Mark only one oval.

- Yes
- No

11. Does this research study present evidence on Metabolic syndrome or risk factors?

Mark only one oval.

- Yes
- No

12. Who is the Screen-er?

Mark only one oval.

- PI
- Co-Screen-er

This content is neither created nor endorsed by Google.

Google Forms

The evidence on the association between FI and Key Metabolic Risk Factors for DSCDs in Sub-Saharan Africa: A Systematic Review

Full Text Screening Level 2 Form (Preliminary Two)

1. Author and Date

2. Title

3. Does this research study present evidence from sub-Saharan Africa?

Mark only one oval.

- Yes
- No

4. Does this research study present evidence on Food Insecurity?

Mark only one oval.

- Yes
- No

5. Does this research study present evidence on Obesity?

Mark only one oval.

- Yes
- No

6. Does this research study present evidence on Diabetes / higher fasting plasma glucose level?

Mark only one oval.

- Yes
- No

7. Does this research study present evidence on Cardiovascular diseases?

Mark only one oval.

- Yes
- No

8. Does this research study present evidence on Dyslipidemia?

Mark only one oval.

- Yes
- No

9. Does this research study present evidence on High Blood Pressure/Hypertension?

Mark only one oval.

- Yes
- No

10. Does this research study present evidence on respiratory diseases/Asthma?

Mark only one oval.

- Yes
- No

11. Does this research study present evidence on stunting/ wasting OR underweight/ Overweight?

Mark only one oval.

- Yes
- No

12. Does this research study present evidence on Metabolic syndrome or risk factors?

Mark only one oval.

- Yes
- No

13. Who is the Screen-er?

Mark only one oval.

- 3rd Co-Screen-er
- PI

This content is neither created nor endorsed by Google.

Google Forms

The evidence on the association between FI and Key Metabolic Risk Factors for DSCDs in Sub-Saharan Africa: A Systematic Review

Data Extraction Form Level 3

1. Author, Year

2. Title

3. Country

4. Geographic setting

5. Study design

6. Study setting

7. Aim of the study

8. Population Size

9. Age (y)

10. Sex (Female/Male, %)?

11. Inclusion & Exclusion Criteria

12. Sample Size

13. Measured Exposure Definition - Food Insecurity

14. Ascertainment of Exposure

15. Number of Exposed Participants

16. Number of Unexposed participants/Comparators Size

17. Definition & Ascertainment of Reported Primary Outcome 1: Obesity

18. Number of exposed participants with the reported outcome

19. Number of unexposed participants with the reported outcome

20. Definition & Ascertainment of Reported Primary Outcome 2: Diabetes

21. Number of exposed participants with the reported outcome

22. Number of unexposed participants with the reported outcome

23. Definition & Ascertainment of Reported Primary Outcome 3: Cardiovascular Diseases

24. Number of exposed participants with the reported outcome

25. Number of unexposed participants with the reported outcome

26. Definition & Ascertainment of Reported Primary Outcome 4: Dyslipidemia

27. Number of exposed participants with the reported outcome

28. Number of unexposed participants with the reported outcome

29. Definition & Ascertainment of Reported Primary Outcome 5: High Blood Pressure - Hypertension?

30. Number of exposed participants with the reported outcome

31. Number of unexposed participants with the reported outcome

32. Definition & Ascertainment of Reported Primary Outcome 6: Underweight?

33. Number of exposed participants with the reported outcome

34. Number of unexposed participants with the reported outcome

35. Definition & Ascertainment of Reported Primary Outcome 7: Overweight?

36. Number of exposed participants with the reported outcome

37. Number of unexposed participants with the reported outcome

38. Definition & Ascertainment of Reported Primary Outcome 8: Other reported metabolic syndrome and outcome of interest

39. Number of exposed participants with the reported outcome

40. Number of unexposed participants with the reported outcome

41. All systematic and random error adjusted?
(e.g. confounding, adjusted covariates, effect medication etc.)

42. Does the research study provide sufficient information for the FI-DSCDs Association

(effect sizes - statistical tool usage)

43. Does the research study provide 'Possible Mechanisms' of the FI-DSCDs Association?

i.e mediating and moderating SDH factors that govern the relationship between FI-DSCDs as presented in the research study

44. Research study key findings

45. Conclusion

46. Notes (Reflection)

This content is neither created nor endorsed by Google.

Google Forms

MIXED METHODS APPRAISAL TOOL (MMAT) VERSION 2018

User guide

Prepared by
Quan Nha HONG[a], Pierre PLUYE[a], Sergi FÀBREGUES[b], Gillian BARTLETT[a], Felicity BOARDMAN[c], Margaret CARGO[d], Pierre DAGENAIS[e], Marie-Pierre GAGNON[f], Frances GRIFFITHS[c], Belinda NICOLAU[a], Alicia O'CATHAIN[g], Marie-Claude ROUSSEAU[h], & Isabelle VEDEL[a]

[a]McGill University, Montréal, Canada; [b]Universitat Oberta de Catalunya, Barcelona, Spain; [c]University of Warwick, Coventry, England; [d]University of Canberra, Canberra, Australia; [e]Université de Sherbrooke, Sherbrooke, Canada; [f]Université Laval, Québec, Canada; [g]University of Sheffield, Sheffield, England; [h]Institut Armand-Frappier Research Centre, Laval, Canada

McGill
Department of Family Medicine
Département de médecine de famille
Academic excellence and innovation in care, teaching and research
Innovation et excellence académique dans les soins, l'enseignement et la recherche

Last update: August 1st, 2018

Part I: Mixed Methods Appraisal Tool (MMAT), version 2018

Category of study designs	Methodological quality criteria	Responses			
		Yes	No	Can't tell	Comments
Screening questions (for all types)	S1. Are there clear research questions?				
	S2. Do the collected data allow to address the research questions?				
	Further appraisal may not be feasible or appropriate when the answer is 'No' or 'Can't tell' to one or both screening questions.				
1. Qualitative	1.1. Is the qualitative approach appropriate to answer the research question?				
	1.2. Are the qualitative data collection methods adequate to address the research question?				
	1.3. Are the findings adequately derived from the data?				
	1.4. Is the interpretation of results sufficiently substantiated by data?				
	1.5. Is there coherence between qualitative data sources, collection, analysis and interpretation?				
2. Quantitative randomized controlled trials	2.1. Is randomization appropriately performed?				
	2.2. Are the groups comparable at baseline?				
	2.3. Are there complete outcome data?				
	2.4. Are outcome assessors blinded to the intervention provided?				
	2.5 Did the participants adhere to the assigned intervention?				
3. Quantitative non-randomized	3.1. Are the participants representative of the target population?				
	3.2. Are measurements appropriate regarding both the outcome and intervention (or exposure)?				
	3.3. Are there complete outcome data?				
	3.4. Are the confounders accounted for in the design and analysis?				
	3.5. During the study period, is the intervention administered (or exposure occurred) as intended?				
4. Quantitative descriptive	4.1. Is the sampling strategy relevant to address the research question?				
	4.2. Is the sample representative of the target population?				
	4.3. Are the measurements appropriate?				
	4.4. Is the risk of nonresponse bias low?				
	4.5. Is the statistical analysis appropriate to answer the research question?				
5. Mixed methods	5.1. Is there an adequate rationale for using a mixed methods design to address the research question?				
	5.2. Are the different components of the study effectively integrated to answer the research question?				
	5.3. Are the outputs of the integration of qualitative and quantitative components adequately interpreted?				
	5.4. Are divergences and inconsistencies between quantitative and qualitative results adequately addressed?				
	5.5. Do the different components of the study adhere to the quality criteria of each tradition of the methods involved?				

UNIVERSITY OF KWAZULU-NATAL
INYUVESI YAKWAZULU-NATALI

Mr Sphamandla Josias Nkambule (210501689)
School Of Nurs & Public Health
Howard College

Dear Mr Sphamandla Josias Nkambule,

Protocol reference number: 00002835
Project title: The Evidence on the Association Between Food Insecurity and Diet-sensitive Chronic Diseases in Sub-Saharan Africa: A Systematic Review and Meta-analysis

Exemption from Ethics Review

In response to your application received on 29 July 2019, your school has indicated that the protocol has been granted **EXEMPTION FROM ETHICS REVIEW.**

Any alteration/s to the exempted research protocol, e.g., Title of the Project, Location of the Study, Research Approach and Methods must be reviewed and approved through an amendment/modification prior to its implementation. The original exemption number must be cited.

For any changes that could result in potential risk, an ethics application including the proposed amendments must be submitted to the relevant UKZN Research Ethics Committee. The original exemption number must be cited.

In case you have further queries, please quote the above reference number.

PLEASE NOTE:
Research data should be securely stored in the discipline/department for a period of 5 years.

I take this opportunity of wishing you everything of the best with your study.

Yours sincerely,

Prof Matlagolo Mosa Moshabela
Academic Leader Research
School Of Nurs & Public Health

UNIVERSITY OF KWAZULU-NATAL

BIOMEDICAL RESEARCH ETHICS COMMITTEE

APPLICATION FOR ETHICS APPROVAL OF AMENDMENTS

NAME OF RESEARCHER: Sphamandla Josias Nkambule

DEPARTMENT: Discipline of Public Health Medicine, School of Nursing and Public Health

TITLE OF STUDY: The Evidence on the Association Between Food Insecurity and Diet-sensitive Chronic Diseases in Sub-Saharan Africa: A Systematic Review and Meta-analysis

ETHICS REFERENCE NO: 00002835

DATE OF ETHICAL APPROVAL OF STUDY: 29 July 2019

DATE OF AMENDMENTS: 15 January 2020

AMENDMENTS REQUESTED:

1. **TITLE OF STUDY:**

   Original protocol states:

   The Evidence on the Association Between Food Insecurity and Diet-sensitive Chronic Diseases in Sub-Saharan Africa: A Systematic Review and Meta analysis

   Amendments Requested:

   Association Between Food Insecurity and Key Metabolic Risk Factors for Diet-Sensitive Non-communicable Diseases in Sub-Saharan Africa: A Systematic Review and Meta-Analysis

Reason for amendment:

For classification purposes and interpretation "metabolic risk factors" is more in line with current literature and diagnostic definition of the study outcomes, when compared to "chronic diseases"

These amendments would not have any impact on participants or patient

1. Itemise required amendments in following format:
   (i) original protocol states..................... amendment requested......... etc.
2. Reason for amendment and the impact this will have on the participant or patient.
3. If additional investigators are added: Outline role and submit 2-page CV and proof of current HPCSA registration and GCP certification with the application.
4. If a new site is added, submit permission letter from the manager of the hospital/clinic/institution, if applicable.

SIGNATURE OF PRINCIPAL RESEARCHER: ....**DATE:** 15/01/2020
